# Ergosterol-Enriched Liposomes with Post-Processing Modifications for Serpylli Herba Polyphenol Delivery: Physicochemical, Stability and Antioxidant Assessment

**DOI:** 10.3390/pharmaceutics17111362

**Published:** 2025-10-22

**Authors:** Aleksandra A. Jovanović, Predrag Petrović, Andrea Pirković, Ninoslav Mitić, Francesca Giampieri, Maurizio Battino, Dragana Dekanski

**Affiliations:** 1 Institute for the Application, Nuclear Energy INEP, University of Belgrade, 11000 Belgrade, Serbia; ajovanovic@inep.co.rs (A.A.J.); andrea.pirkovic@inep.co.rs (A.P.); ninoslavm@inep.co.rs (N.M.); dragana.dekanski@inep.co.rs (D.D.); 2 Innovation Center, the Faculty of Technology and Metallurgy, University of Belgrade, 11000 Belgrade, Serbia; 3 Department of Clinical Sciences, Università Politecnica delle Marche, 60131 Ancona, Italy; f.giampieri@univpm.it; 4 Joint Laboratory on Food Science, Nutrition, and Intelligent Processing of Foods, Polytechnic University of Marche, Italy, Universidad Europea del Atlántico, Spain, and Jiangsu University, China, Università Politecnica delle Marche, 60131 Ancona, Italy; 5 Research Group on Food, Nutritional Biochemistry and Health, Universidad Europea del Atlántico, Isabel Torres 21, 39011 Santander, Spain; 6 International Joint Research Laboratory of Intelligent Agriculture and Agri-Products Processing, Jiangsu University, Zhenjiang 212013, China

**Keywords:** ergosterol, liposomes, polyphenol antioxidants, rheology, stability, Serpylli herba

## Abstract

**Background/Objectives:** In the present study, ergosterol, a novel natural and animal-free alternative sterol, was investigated, and its effects on liposomal properties were assessed. Importantly, ergosterol’s fungal origin offers a sustainable substitute for cholesterol, aligning with current trends in natural and vegan-friendly formulations. **Methods:** This study explored the effect of ergosterol content (10 mol% vs. 20 mol%) on the encapsulation efficiency (EE), physical properties, morphology, antioxidant activity, lipid peroxidation, and storage stability of Serpylli herba extract-loaded liposomes. **Results:** Liposomes with 20 mol% ergosterol exhibited significantly higher EE (~81.0%) than those with 10 mol% (~75.6%), along with improved resistance to UV- and freeze-drying-induced reduction in EE. Extract loading resulted in a reduced particle size, indicating favorable bilayer interactions, whereas lyophilization increased size and polydispersity, reflecting structural destabilization. However, 20 mol% ergosterol improved vesicle uniformity and surface charge stability, suggesting enhanced bilayer rigidity. Zeta potential and mobility trends supported improved colloidal stability in ergosterol-enriched systems under all tested conditions. Over 28 days at 4 °C, non-treated extract-loaded liposomes with a higher ergosterol content demonstrated enhanced vesicle integrity. During storage, UV-treated and lyophilized liposomes with 20 mol% ergosterol maintained more consistent size and charge profiles, indicating better membrane reorganization and stability. Nanoparticle tracking analysis demonstrated that ergosterol content modulates vesicle concentration in a dose-dependent manner, highlighting the role of membrane composition in liposome formation and potential dose uniformity. Transmission electron microscopy analysis of extract-loaded liposomes demonstrated well-defined vesicles with intact structural features. A study in a Franz diffusion cell revealed that ergosterol-enriched liposomes significantly delayed polyphenol release compared to free extract, confirming their potential for controlled delivery. Antioxidant activity was preserved in all liposomal systems, with higher ergosterol content supporting improved ABTS radical scavenging potential after stress treatments. FRAP assay results remained stable across formulations, with no major differences between sterol levels. TBARS analysis demonstrated that Serpylli herba extract significantly reduced UV-induced lipid peroxidation in ergosterol-enriched liposomes, underscoring its protective antioxidant role. **Conclusions:** Higher ergosterol content enhanced liposomal performance in terms of encapsulation, structural resilience, and antioxidant retention, particularly under UV and lyophilization stress. Ergosterol-containing liposomes exhibited improved stability, favorable particle size distribution, and high encapsulation efficiency, while maintaining the antioxidant functionality of the incorporated Serpylli herba polyphenol-rich extract. These findings highlight the potential of ergosterol-based liposomes as robust carriers for bioactive compounds in pharmaceutical and nutraceutical applications that align with current trends in green and vegan-friendly formulations.

## 1. Introduction

*Thymus serpyllum* L., commonly known as wild thyme or creeping thyme, is a perennial herbaceous plant belonging to the Lamiaceae family. Its low-growing, creeping habit is characterized by woody stems, small aromatic leaves, and clusters of tubular violet to pink flowers. This species naturally thrives in temperate regions, often found in dry, rocky soils, grasslands, and open woodland habitats across Europe, Asia, and parts of North America [1,2,3]. The essential oil and extracts derived from *T. serpyllum* are rich in bioactive constituents, notably polyphenols, such as flavonoids, and phenolic acids, along with terpenoids [1,2]. These compounds confer a broad spectrum of biological activities, including antioxidant, antimicrobial, anti-inflammatory, and potential anticancer effects [1,4,5]. Serpylli herba (aerial part of the plant), derived from *T. serpyllum*, is widely recognized for its rich content of bioactive compounds, including essential oil, flavonoids, and phenolic acids, which contribute to its potent antioxidant and antimicrobial properties [5]. Traditionally used in herbal medicine, Serpylli herba exhibits various pharmacological effects, including anti-inflammatory, spasmolytic, and expectorant activities, which support its application in respiratory, gastrointestinal, and urogenital disorders [5,6,7,8]. The literature highlighted its potential as a natural preservative and functional ingredient in various formulations due to its safety profile and diverse biological effects [5].

Although numerous preclinical studies have highlighted the potential health benefits of dietary polyphenols, issues such as chemical instability, low bioavailability, and unfavorable sensory properties (notably the bitterness) have restricted their broader incorporation into food functional products or supplements [9]. Polyphenols are also inherently unstable, being susceptible to degradation triggered by environmental factors such as light, oxygen, pH fluctuations, and temperature during processing and storage [10,11]. Moreover, their oral bioavailability is low due to poor absorption, rapid metabolism, and elimination in the gastrointestinal tract [11,12]. The pharmacokinetic challenges, along with poor solubility, limited stability, and the lack of targeted delivery, continue to hinder the effective application of polyphenol extracts in therapeutic settings [13,14]. These limitations justify the development of encapsulation strategies to protect polyphenols from degradation, enhance their bioavailability, and mask undesirable flavors [11,15]. Liposomal encapsulation, for instance, can effectively improve the stability and controlled release of extracted Serpylli herba polyphenols, consequently optimizing their therapeutic potential while minimizing sensory drawbacks.

Therefore, in response to the challenges associated with the instability and limited bioavailability of plant bioactive compounds, the present research has focused on advanced formulation strategies, particularly the use of liposomes as biocompatible nanocarriers capable of enhancing compound stability, permeability, and controlled release. Among various lipid-based delivery systems, liposomal vesicles have emerged as one of the most widely applied platforms in biomedical and pharmaceutical applications due to their structural versatility and favorable biophysical properties [16]. These carriers consist of spherical bilayer membranes that offer both hydrophilic and lipophilic microenvironments, facilitating the encapsulation of a broad spectrum of active substances [17]. Moreover, the flexibility of liposomes, including modifiable size, lamellarity, and surface characteristics, combined with their biodegradability and safety, makes them ideal candidates for drug delivery applications, particularly for plant-derived bioactives [18,19,20]. In this context, polyphenolic compounds from botanical sources particularly benefit from liposomal encapsulation, which enhances their absorption, bioavailability, and protection from environmental degradation [21,22,23]. Beyond protection, liposomes also play a crucial role in optimizing therapeutic efficacy by minimizing toxicity, reducing adverse effects, and promoting targeted and sustained delivery of the encapsulated agents [24,25].

The incorporation of sterols into liposomal formulations is a well-established approach for modifying bilayer properties, particularly membrane fluidity, permeability, and overall pharmacokinetic behavior of the encapsulated compounds [26,27,28,29]. In conventional liposomal systems, cholesterol is widely applied as a bilayer-stabilizing sterol. However, ergosterol possesses unique structural features, including additional double bonds and a conjugated sterol ring, which affect membrane packing and order. These molecular differences can enhance bilayer rigidity, while simultaneously improving permeability control and oxidative resistance compared with cholesterol [30,31,32,33]. Among sterols, ergosterol, a fungal-derived sterol, has garnered attention for its capacity to enhance membrane packing density and contribute to the physical stability of liposomes, thereby improving the performance and robustness of the delivery system [30,31]. Unlike cholesterol, ergosterol possesses a higher degree of unsaturation, which influences its interaction with phospholipid membranes. While it still contributes to membrane ordering, it does so less rigidly than cholesterol, thereby maintaining a more fluid bilayer environment in comparison to cholesterol [34]. Studies have shown that ergosterol-containing liposomes exhibit a different biophysical behavior compared to their cholesterol-containing counterparts. For instance, the reduced membrane ordering observed in ergosterol-based systems can facilitate better homogeneity, particularly in the presence of charged lipids, as highlighted by Yoda [35] and Song et al. [36]. This sterol also appears to mitigate the sensitivity of the bilayer to charged environments, whereas cholesterol-containing membranes tend to respond more strongly to electrostatic interactions [35]. Therefore, per os administration of ergosterol-containing liposomes can show promising results in protecting sensitive molecules from gastrointestinal degradation and facilitating controlled release. Beyond its structural role, ergosterol has been explored for its multiple health benefits (such as anti-inflammatory, antioxidant, and anticancer activities), particularly in formulations targeting cardiovascular, hepatic, and immune health, due to its lipid-lowering properties, including reductions in serum cholesterol and triglyceride levels [37,38]. Thus, the combination of ergosterol’s bioactivity and its stabilizing effect in liposomal carriers presents an innovative approach to improve oral bioavailability and therapeutic efficacy of encapsulated agents. Furthermore, ergosterol can be obtained from fungi and yeast, providing a natural, sustainable, and animal-free alternative that aligns with current trends in green and vegan-friendly formulations. These aspects highlight the potential of ergosterol as a novel functional substitute for cholesterol in liposomal carriers.

In the present study, ergosterol–phospholipid liposomes, as the carrier for Serpylli herba polyphenol extract that we have characterized previously [3], were developed, followed by post-preparation modifications (UV irradiation and lyophilization). Specifically, the encapsulation efficiency (EE), antioxidant capacity (ABTS, DPPH, FRAP, and TBARS assays), particle size, polydispersity index (PDI), zeta potential, mobility, density, surface tension, viscosity, stability during storage, and stability after UV irradiation and lyophilization of the obtained liposomes were investigated. Furthermore, nanoparticle tracking analysis (NTA), transmission electron microscopy (TEM), and study of the release kinetics were performed as well. By integrating Serpylli herba extract into ergosterol-based liposomes, this study addresses both the instability of plant polyphenols and the need for sustainable sterol substitutes in pharmaceutical and nutraceutical formulations.

## 2. Materials and Methods

### 2.1. Plant Material and Chemicals

Serpylli herba was from the Institute for Medicinal Plants Research “Dr Josif Pančić”, Pančevo, Serbia. The following reagents were used: ethanol and sodium carbonate (Fisher Scientific, Loughborough, Leicestershire, UK), Folin–Ciocalteu reagent and gallic acid (Merck, Saint Louis, MO, USA), potassium persulfate (Centrohem, Stara Pazova, Serbia), Phospholipon^®^ 90G (unsaturated diacyl-phosphatidylcholine) (Lipoid GmbH, Ludwigshafen, Rhineland-Palatinate, Germany), 2,2′-azino-bis(3-ethylbenzothiazoline-6-sulphonic acid)—ABTS, 2,2-diphenyl-1-picrylhydrazyl-DPPH, 2,4,6- tripyridy-s-triazine—TPTZ, ferric chloride, sulfuric acid, hydrochloric acid, acetic acid, sodium acetate, and ergosterol (Sigma-Aldrich, Saint Louis, MO, USA). Thiobarbituric acid, trichloroacetic acid, perchloric acid, paraformaldehyde, and glutaraldehyde were from Sigma-Aldrich (Darmstadt, Germany). Phospholipon^®^ 90G is a purified natural phospholipid preparation obtained from soy lecithin, consisting mainly of >90% phosphatidylcholine, with minor fractions of other phospholipids, such as phosphatidylethanolamine and phosphatidylinositol. The predominant phospholipid, phosphatidylcholine, is electrically neutral at physiological pH, while trace components may carry weakly negative charges. With respect to the phase state, Phospholipon^®^ 90G is considered a hydrogenated phospholipid mixture that exhibits a relatively high phase transition temperature (gel-to-liquid crystalline transition, ~55–60 °C), leading to a more rigid bilayer structure compared with unsaturated lecithin.

### 2.2. Preparation of Serpylli Herba Extract

Serpylli herba liquid extract was prepared by using 0.3 mm particle size of plant material (air-dried aerial part of wild thyme). In addition, 50% *v*/*v* ethanol, as an extraction medium and 30:1 solvent-to-solid ratio (mL/g) were employed. The extraction procedure was undertaken for 15 min at 80 °C, in the incubator shaker (KS 4000i control, IKA, Staufen im Breisgau, Baden-Württemberg, Germany). The extract preparation was performed according to a previously optimized extraction protocol [3]. The obtained extract was used for the liposome preparation, and the rest of the extract was stored at 4 °C until further analysis.

### 2.3. Preparation of Serpylli Herba Extract-Loaded Liposomes

Ergosterol–phospholipid liposomes containing Serpylli herba extract were prepared using the proliposome method [39]. Liposomal particles were prepared using a commercial mixture of phospholipids (Phospholipon^®^ 90G) and ergosterol (10 mol% or 20 mol%). Ergosterol was incorporated at 10% and 20% molar ratios relative to phospholipids, as concentrations within this range have been shown to maximize sterol-phospholipid interactions and improve bilayer stability without promoting sterol crystallization or phase separation [40]. A mixture of phospholipids and ergosterol (1 g) and ethanol Serpylli herba extract (4 mL) was stirred at 50 °C for 30 min in an uncovered beaker to evaporate ethanol and obtain a homogenous mixture. Ethanol evaporation was performed at 50 °C, a temperature sufficient for complete solvent removal, while minimizing the risk of oxidative or thermal degradation of both phospholipids and the encapsulated Serpilly herba polyphenols (the extract was prepared at 80 °C as stated in Section 2.2). After cooling to room temperature, the aqueous phase (20 mL of ultrapure water) was added in small portions to promote controlled hydration of the lipid film and to facilitate the gradual self-assembly of liposomes. A stepwise addition helps to avoid local lipid oversaturation and prevents the formation of large aggregates or multilamellar structures. By allowing lipids to reorganize progressively upon hydration, this method enhances the homogeneity of vesicle formation, leading to liposomes with a more uniform size distribution and lower polydispersity. Furthermore, the controlled hydration reduces mechanical stress on the bilayer and improves the encapsulation efficiency of hydrophilic bioactives present in the aqueous medium. Subsequently, the dispersion was stirred at 800 rpm for 1 h at ambient temperature. Plain liposomes (without extract) were prepared as a control, but instead of Serpylli herba extract, the same amount of 50% *v*/*v* ethanol (4 mL) was added. The liposomes were kept at 4 °C until future analyses.

### 2.4. Encapsulation Efficiency

EE was determined by using the indirect method. EE was calculated by the content of polyphenols in the supernatant as shown in Equation (1):

(1) $$EE\;(\%)=\;\frac{TPCi-TPCsup}{TPCi}\;\times 100$$

where TPC_i_ is the initial content of total polyphenols in Serpylli herba extract used for the preparation of liposomes, and TPC_sup_ is the content of total polyphenols determined in the supernatant.

Free Serpylli herba extract was removed from liposomal suspension by centrifugation at 17,500 rpm and 4 °C for 45 min in a Thermo Scientific Sorval WX Ultra series ultracentrifuge (Thermo Fisher Scientific, Waltham, MA, USA). The total polyphenol content (TPC) in the extract and supernatants was determined using the modified Folin–Ciocalteu method with absorbance readings at 765 nm [3].

### 2.5. Size, PDI, Zeta Potential, and Mobility Analyses of the Liposomes

The size, PDI, zeta potential, and mobility of unloaded and Serpylli herba extract-loaded liposomes (immediately after liposome preparation, UV irradiation, lyophilization, and during the 28-day storage study) were determined using photon correlation spectroscopy (PCS) in Zetasizer Nano Series, Nano ZS (Malvern Instruments Ltd., Malvern, Worcestershire, UK). Electrophoretic mobility values were automatically converted to zeta potential using the instrument software. Namely, the used Zetasizer Nano series calculates the zeta potential by determining the electrophoretic mobility and then applying the Henry equation. Each sample was diluted 200 times, and a volume of 1 mL was measured three times at room temperature.

### 2.6. Analyses of the Density, Surface Tension, and Viscosity of the Liposomes

Density and surface tension of non-treated and UV-irradiated ergosterol–phospholipid liposomes (empty and loaded with Serpylli herba extract) were determined using a silicon crystal as the immersion body and a Wilhelmy plate, respectively, (Force Tensiometer K20, Kruss, Hamburg, Germany). Each liposomal sample (20 mL) was examined three times at room temperature.

The viscosity of non-treated and UV-irradiated ergosterol–phospholipid liposomes (unloaded and extract-loaded samples) was determined using the Rotavisc lo-vi device equipment with VOL-C-RTD chamber, VOLS-1 adapter, and spindle (IKA, Staufen im Breisgau, Germany). Each sample (6.7 mL) was examined three times at room temperature.

### 2.7. Stability Study

#### 2.7.1. Storage Stability Study

The measurements of particle size, PDI, zeta potential, and mobility of liposomes were repeated on the 1st, 7th, 14th, 21st, and 28th days after the liposomal preparation to monitor the stability of the liposomal system. During the stability study, the liposomes were stored in the refrigerator at 4 °C. Refrigerated conditions were chosen to mimic typical storage environments for liposomal formulations, ensuring that the data obtained are relevant for practical applications. The prepared liposomal samples were stored at 4 °C in tightly sealed Eppendorf plastic vials (Eppendorf AG, Hamburg, Germany). Prior to sealing, nitrogen gas was carefully introduced into the system to establish an inert atmosphere. Additionally, the vials were further wrapped with Parafilm^®^ (Bemis, Neenah, WI, USA) to minimize oxygen penetration during storage. This combined approach was applied to reduce the likelihood of oxidative degradation and the formation of lipid peroxidation products.

#### 2.7.2. UV-Stability Study

To investigate changes in liposomes after exposure to UV irradiation, which is applied in the food, pharmaceutical, and cosmetic industries for sterilization, the liposomes (3 mL, unloaded and extract-loaded) were exposed to UV-C irradiation [41]. Exposure to 253.7 nm UV-C for 20 min in a thin layer allowed controlled evaluation of the stability of liposomes under conditions relevant to practical applications. Particle size, PDI, zeta potential, mobility, density, surface tension, viscosity, and antioxidant capacity of liposomes, as well as TPC in supernatants and release kinetics, were analyzed immediately after UV irradiation to assess the direct effects of UV stress. Monitoring changes in particle size, PDI, zeta potential, and mobility during 28 days of refrigerated storage enabled evaluation of the long-term impact of UV exposure on liposome integrity. This approach provides insight into the resilience of ergosterol-containing liposomes and the potential protective effect of encapsulated polyphenols under sterilization-relevant conditions.

#### 2.7.3. Stability Study After Lyophilization

It is known that lyophilized products are stable over long periods, due to the prevention of hydrolytic and oxidative degradation of active components during storage. Thus, in the present study, the changes in liposomal characteristics after lyophilization were investigated. Cryoprotectants such as sugars (e.g., sucrose, trehalose) are often included to prevent vesicle aggregation and membrane fusion during freeze-drying; however, in the present study, they were intentionally omitted. The rationale was twofold: (i) to specifically assess the intrinsic stabilizing capacity of ergosterol within the bilayer, without interference from external stabilizers, and (ii) to avoid possible interactions between added cryoprotectants and polyphenols that could complicate interpretation of the results related to EE and antioxidant assays. Indeed, freshly prepared empty and extract-loaded liposomes (2 mL) were centrifuged, the supernatant was discarded, and the pellet was dispersed in 2 mL of ultrapure water. The centrifugation and resuspension steps before lyophilization were performed to concentrate the liposomal formulations and remove unencapsulated components, ensuring that subsequent analyses reflect the properties of the vesicle-encapsulated fraction. The samples were lyophilized at −75 °C and 0.011 mbar for 24 h in the Beta 2–8 LD plus device (Christ, Osterode am Harz, Germany). Lyophilization at the mentioned conditions was employed to remove water under mild conditions, thereby minimizing hydrolytic and oxidative degradation of both the lipids and encapsulated polyphenols. The lyophilized liposomes were then reconstructed with ultrapure water to their original volume before further analyses (PCS, antioxidant potential, and TPC measurements). Reconstitution to the original volume with ultrapure water allowed the liposomes to recover their dispersion state, enabling accurate measurements. This procedure ensures that observed changes in liposomal characteristics are attributable to the effects of lyophilization itself rather than dilution, handling, or residual free extract and provides a controlled approach to evaluate storage stability. In addition, alterations in vesicle size, PDI, zeta potential, and mobility of the lyophilized liposomes were monitored during 28 days of storage in the refrigerator. Monitoring the mentioned variables during refrigerated storage provides essential information about the physical stability of the lyophilized liposomes. These parameters directly reflect potential aggregation, fusion, or structural reorganization of vesicles after rehydration. Tracking their evolution over 28 days enables the assessment of shelf-life, the effectiveness of the lyophilization process, and the potential ability of ergosterol to preserve vesicle integrity.

### 2.8. Nanoparticle Tracking Analysis

The concentration and size distribution of Serpylli herba extract-loaded liposomes with ergosterol (10 mol% and 20 mol%) were analyzed using a ZetaView^®^ QUATT PMX-430 nanoparticle tracking analyzer (Particle Metrix, Inning am Ammersee, Germany) with ZetaView software version 8.05.16 SP3. The instrument was automatically checked, aligned, and calibrated with 100 nm polystyrene beads before measurements. Samples were diluted in ultrapure water, and measurements were performed in light scatter mode (488 nm laser) at a shutter speed of 130, frame rate of 30 fps, and sensitivity of 78. Post-acquisition settings were a minimum area of 10, a maximum area of 1000, and a brightness threshold of 30. Each sample was measured in triplicate at up to 11 positions, with thorough cleaning between runs.

### 2.9. Transmission Electron Microscopy

The morphology of Serpylli herba extract-loaded liposomes with ergosterol (10 mol% and 20 mol%) was examined by TEM. Briefly, 10 µL of each sample was placed onto 200-mesh formvar-coated copper grids and allowed to adsorb at room temperature for 30 min. Grids were fixed in 2% paraformaldehyde for 10 min, rinsed three times with ultrapure water, and subsequently treated with 2.5% glutaraldehyde for 5 min. After a final rinse with ultrapure water, the samples were air-dried and imaged using a Philips CM12 TEM (Philips, Eindhoven, The Netherlands).

### 2.10. Polyphenol Release Study

The release of polyphenols from pure extract and extract-loaded ergosterol–phospholipid liposomes (ergo 20%, because of higher EE, non-treated and UV-irradiated) was studied for 24 h, at 25 °C in water in a Franz diffusion cell using an acetate-cellulose membrane. Since the concentrations of polyphenols were very low in the receptor medium and it was impossible to quantify them in the Folin–Ciocalteu method, the content of polyphenols in the samples was determined directly spectrophotometrically at 320 nm.

### 2.11. Antioxidant Assays

The antioxidant potential of Serpylli herba extract-loaded liposomes (non-treated, UV-irradiated, and lyophilized samples) was analyzed according to the previously published assays, ABTS and DPPH radical scavenging methods [42], and ferric reducing antioxidant power, i.e., FRAP assay [43]. Additionally, lipid peroxidation of liposomal formulations was investigated using the TBARS (thiobarbituric acid reactive substances) assay [44]. Since the mentioned assays are based on different mechanisms, they are used to obtain a complete insight into the antioxidant capacity or lipid peroxidation of the produced liposomes. Liposomes with extract were centrifuged before antioxidant assays to eliminate the impact of non-encapsulated antioxidant compounds from the extract.

#### 2.11.1. ABTS Assay

The absorbance of an ethanol solution of ABTS radicals (previously activated by potassium persulfate) in a volume of 2 mL and a liposomal sample (20 µL) was spectrophotometrically measured at 734 nm. The measurement was performed after 6 min of incubation in the dark at room temperature, and the antioxidant potential was calculated as:

(2) $$\%\;of\;neutralization\;=\;\frac{\left(A_{0ABTS}-A_{x}\right)}{A_{0ABTS}}\;\times 100\;$$

where A_0ABTS_ was the absorbance of the control (ABTS ethanol solution and water, ~0.700) and A_x_ was the absorbance of the sample.

#### 2.11.2. DPPH Assay

The absorbance of an ethanol solution of DPPH radicals (2 mL) and liposomal sample (20 µL) was spectrophotometrically measured at 517 nm. The measurement was performed after 20 min of incubation in the dark at room temperature, and the antioxidant potential was calculated as:

(3) $$\%\;of\;neutralization\;=\;\frac{\left(A_{0DPPH}-A_{x}\right)}{A_{0DPPH}}\;\times 100\;$$

where A_0DDPH_ was the absorbance of the control (DPPH ethanol solution and water, ~0.800) and A_x_ was the absorbance of the sample.

#### 2.11.3. FRAP Assay

The FRAP reagent contained 2.5 mL of a 10 mmol/L TPTZ solution in 40 mmol/L HCl, 2.5 mL of 20 mmol/L FeCl_3_, and 25 mL of 0.3 mol/L acetate buffer (pH of 3.6). It was prepared freshly and warmed at 37 °C. The liposomes (40 µL) were mixed with ultrapure water (0.2 mL) and FRAP reagent (1.8 mL). The mixture was incubated in the dark at 37 °C for 10 min. Subsequently, the mixture was centrifuged at 17,500 rpm for 15 min. The absorbance of the reaction mixture was measured spectrophotometrically at 593 nm. The 1 mmol/L of FeSO_4_ was used as the standard solution. The results were expressed as the concentration of antioxidant compounds having a ferric reducing ability equivalent to that of 0.5 mmol/L of FeSO_4_.

#### 2.11.4. TBARS Assay

Lipid peroxidation of liposomal formulations containing Serpylli herba extract and 10 mol% or 20 mol% of ergosterol was assessed using the TBARS assay, with plain liposomes serving as the control. Both control and extract-loaded liposomes were subjected to UV irradiation for 12 h, while parallel samples from the same batch were stored in the dark and used as non-irradiated controls. At predetermined time points (1, 3, 5, 7, and 12 h), 100 μL aliquots of the liposome dispersions were withdrawn for analysis. Each aliquot was combined with 1.5 mL of 20% trichloroacetic acid and 1 mL of a stock mixture containing 2% thiobarbituric acid and 20% perchloric acid (1:1, *v*/*v*) in glass tubes. The samples were heated at 100 °C for 25 min in a water bath (Thermo Scientific Precision GP 10, Thermo Fisher Scientific, Waltham, MA, USA). Then, the samples were rapidly cooled in ice water to terminate the reaction. Precipitates were removed by centrifugation (3000 rpm, 8 min). The absorbance of the resulting pink supernatant, generated from the reaction of lipid hydroperoxides with thiobarbituric acid, was recorded at 532 nm.

The absorbance of all samples and analyses was read on a UV spectrophotometer, UV-1800 (Shimadzu, Kyoto, Japan).

### 2.12. Statistical Analysis

In the present study, the statistical analysis was performed by using analysis of variance (one-way ANOVA) followed by Duncan’s post hoc test within the statistical software, STATISTICA 7.0 (StatSoft, Tulsa, OK, USA). The chosen tests were selected based on the normality of data distribution and homogeneity of variance. Before applying parametric tests, data was assessed for normality using the Shapiro–Wilk test and for homogeneity of variance using Levene’s test. Duncan’s post hoc test was used due to its higher sensitivity in detecting differences among multiple formulations. This test is especially useful in formulation studies where subtle changes in composition can yield meaningful performance shifts. Additionally, 95% confidence intervals were shown to provide a more robust assessment of the magnitude and reliability of the observed differences (Supplementary Material, Tables S1–S3). The differences were considered statistically significant at *p* < 0.05, *n* = 3.

## 3. Results and Discussion

### 3.1. Encapsulation Efficiency of the Liposomes

EE is a crucial parameter reflecting the capacity of liposomal systems to retain bioactive compounds within their bilayers or aqueous cores [45]. The mentioned parameter is measured immediately after the liposomal preparation, UV irradiation, and lyophilization. The results are shown in Table 1, while 95% confidence intervals related to the data analyzed are shown in Table S1.

As can be seen in Table 1, liposomes containing 20 mol% of ergosterol showed significantly higher EE of polyphenols, compared to samples with 10 mol% of ergosterol. Specifically, non-treated liposomes containing 10 mol% ergosterol achieved an EE of ~75.6%, while the 20 mol% ergosterol formulation exhibited a significantly higher EE of ~81.0%. This enhancement suggests that increased ergosterol content strengthens the lipid bilayer, reducing membrane permeability and thus improving retention of the extract. Sterols, including cholesterol, β-sitosterol, and ergosterol, are usually part of the lipid composition of liposomes, with the role to modulate membrane fluidity, promote stability of the lipid bilayer, and prevent the leakage of the encapsulated components [30,31,40]. Since sterols are in the lipid bilayer and the sterol carbohydrate tail connects with the hydrophobic fatty acyl chains, the sterol hydroxyl group interacts with the hydrophilic head group of lipids. It leads to a more ordered liposomal membrane and the restriction of acid chains’ movement. Apart from that, the increase in sterol concentration resulted in increased packing, bilayer cohesion, and mechanical stiffness, and decreased membrane permeability and mobility of the carbohydrate chains [40,46]. Thus, due to the mentioned facts, it is expected that the liposomes with a higher content of ergosterol would show a higher content of encapsulated polyphenols. Following UV irradiation, a statistically significant decrease in EE was observed for 10 mol% ergosterol liposomes (~73.9%). However, 20 mol% ergosterol formulation maintained its high encapsulation efficiency (~81.5%) (Table 1). Namely, UV irradiation did not cause polyphenol leakage from the 20 mol% ergosterol liposomes, i.e., reduction in EE. These results indicate that higher ergosterol concentrations confer resistance to photo-induced bilayer destabilization, preserving liposomal integrity under UV stress. After the lyophilization process, EE values were statistically significantly lower than in the case of both non-treated parallels (Table 1). Lyophilization notably affected EE values, with a marked reduction in 10 mol% ergosterol liposomes (EE of ~49.4%), likely due to structural disruptions during freeze-drying. Conversely, 20 mol% ergosterol liposomes better retained encapsulated compounds post lyophilization, displaying an EE of ~70.4%. This finding emphasizes the protective role of ergosterol in maintaining liposomal stability during dehydration and rehydration cycles. The results obtained for lyophilized samples were expected since there were no added cryoprotectants to protect the liposomal membrane from freezing damage, and thus to prevent polyphenol leakage. The data demonstrate that the incorporation of a higher content of ergosterol improves EE and stabilizes liposomes against environmental stresses, highlighting its utility in optimizing delivery systems for polyphenol-rich extracts.

### 3.2. Liposome Size, PDI, Zeta Potential, and Mobility

The size, PDI, zeta potential, and mobility of liposomes formulated with varying ergosterol content and extract loading were assessed after liposome preparation and under UV-irradiation and lyophilization conditions. These parameters critically influence liposomal stability, dispersity, and behavior in biological systems. Thus, the influence of extract, different ergosterol contents, and post-preparation modifications on mentioned variables was examined using PCS. The results are shown in Table 1 (the values measured after the preparation of liposomes, UV irradiation, and lyophilization). 95% confidence intervals related to the analyzed data are shown in Table S1.

As can be seen in Table 1, the diameter of plain ergosterol-containing liposomes was notably larger than the diameter of their extract-loaded counterparts, regardless of ergosterol concentration. It indicates that extract presence may promote the formation of smaller vesicles, possibly by modulating lipid packing. Specifically, non-treated plain liposomes containing 10 mol% of ergosterol exhibited a size of ~604.7 nm, which was significantly reduced to ~460.0 nm upon encapsulation of the extract. A similar trend was observed for liposomes with 20 mol% of ergosterol, where size decreased from ~596.0 nm to ~445.0 nm after extract loading. These results suggest that the presence of bioactive plant compounds may influence lipid packing and bilayer curvature during vesicle formation, leading to smaller and more compact vesicles. Since polyphenol compounds are usually positioned in the bilayer, they reduce the number of incorporated sterols and thus provide the formation of smaller liposome vesicles [47]. Such behavior has been attributed to interactions between polyphenolic compounds and phospholipid headgroups, which can modulate membrane rigidity and facilitate the formation of smaller unilamellar vesicles rather than multilamellar ones [48]. Additionally, flavonoids may act as natural surfactants, enhancing bilayer flexibility and preventing excessive vesicle growth during the hydration step [49]. Although literature data show that a higher concentration of sterols causes an increase in liposomal size due to the interactions between phospholipids and sterols, and formation of inter-lipid space [50], in the case of ergosterol–phospholipid liposomes, this did not occur (Table 1). Perhaps a further increase in ergosterol concentration (over 20 mol%) would show a statistically significant difference in particle size. However, these liposomes, although prepared (30 mol% and 40 mol% of ergosterol), were not taken for further analyses, because of their instability and ergosterol precipitation after liposome centrifugation. Hence, between liposomes containing 10 mol% and 20 mol% of ergosterol (plain and extract-loaded), there were no statistically significant differences in particle size. The vesicle size of both plain and extract-loaded liposomes remained relatively unchanged following UV irradiation. It indicates a notable resistance of the liposomal bilayers to photo-induced structural disruption. For instance, plain liposomes containing 10 mol% and 20 mol% of ergosterol exhibited sizes of ~602.5 nm and ~586.0 nm post irradiation, respectively, with no statistically significant differences compared to non-treated parallels (Table 1). Similarly, extract-loaded liposomes maintained consistent vesicle dimensions, with ~451.3 nm and ~454.3 nm after UV exposure for 10 mol% and 20 mol% ergosterol formulations, respectively. This stability may be attributed to the incorporation of ergosterol, which enhances bilayer packing and reduces membrane permeability. This thereby mitigates UV-induced oxidative damage and structural deformation [32]. Furthermore, the presence of polyphenolics might provide an additional protective effect through their radical scavenging activity, counteracting UV-induced lipid peroxidation and maintaining vesicle integrity [51]. These findings highlight the role of ergosterol and polyphenolic constituents in preserving liposomal architecture under stress conditions. Lyophilization led to a notable increase in vesicle size across all formulations. The lyophilization caused a statistically significant increase in vesicle size (5.5–8% for empty liposomes and ~22.5% for extract-loaded liposomes) (Table 1). In plain liposomes (10 mol% and 20 mol% of ergosterol), average diameters increased to ~637.5 nm and ~644.3 nm, respectively. It indicates structural expansion likely caused by stress during freeze-drying and subsequent rehydration. Similarly, extract-loaded liposomes also showed significant enlargement, with sizes rising to ~539.7 nm and ~544.3 nm. It suggests that although the presence of polyphenols mitigated dehydration-induced shrinkage to some extent, it could not entirely prevent membrane perturbation and vesicle swelling. Namely, lyophilized liposomes exhibited the largest particle sizes due to aggregation or fusion events during freeze-drying and rehydration, which can be partially mitigated by ergosterol enrichment. This increase may result from vesicle fusion or partial disruption of bilayer integrity during lyophilization-rehydration cycles, a phenomenon commonly reported for liposomes lacking cryoprotectants [52]. According to literature data, lyophilization causes an increase in liposome size, even 2.5–3.5 times [53,54].

PDI values, as a measure of particle size distribution in liposomal dispersion, are also presented in Table 1. Measured PDI values reflected various size distributions, with non-treated liposomes showing relatively low PDI, indicating moderate monodispersity. Additionally, non-treated liposomes with extract possessed higher PDI, i.e., higher heterogeneity (0.355–0.389), in comparison to plain counterparts (0.235–0.276). This increase can be attributed to several factors, primarily the complex phytochemical composition of the plant extract, which likely affects lipid packing and vesicle formation dynamics. Plant extracts contain a diverse array of bioactive molecules, including phenolic acids, flavonoids, and other amphiphilic or hydrophilic compounds, which can interact differently with the lipid bilayer during vesicle formation. It promotes the development of different populations of vesicles, i.e., a higher heterogeneity [18,55]. A similar trend was reported by Baranauskaite et al. [56]. They observed that the encapsulation of polyphenol-rich oregano extracts into liposomes increased PDI due to the interference of extract constituents with vesicle formation and stability. These findings are consistent with the notion that the interactions between plant-based compounds and liposomes are governed by chemical affinity and physical and structural properties of both the lipid bilayer and the encapsulated compounds [57]. Nevertheless, in lipid-based drug delivery systems, including liposomes and nanoliposomes, a PDI of ≤0.3 is regarded as indicative of a uniform vesicle size distribution and acceptable formulation homogeneity [58]. Additionally, non-treated empty liposomes with 20 mol% of ergosterol have a higher PDI compared to the sample with 10 mol% of ergosterol. According to Zhao et al. [59], a higher amount of sterol causes an increase in heterogeneity. The obtained PDI values of UV-treated liposomes (0.251–0.387) indicate the existence of a moderately uniform system within both unloaded and extract-loaded liposomes as well. Lyophilized samples exhibited significantly increased PDI values (up to ~0.857), highlighting destabilization and heterogeneity induced by processing stresses and suggesting partial disruption of vesicle integrity [56].

The zeta potential of empty liposomes and liposomes with extract is shown in Table 1 as well. Zeta potential values were consistently negative across all samples. It ranges from ~−22.5 mV to ~−28.9 mV, with variations attributed to ergosterol content, extract presence, and post-preparation modifications. Non-treated liposomes containing 20 mol% of ergosterol had a significantly lower absolute value of zeta potential (~−24.3 mV for plain and ~−22.5 mV for extract-loaded samples) than liposomes with 10 mol% of ergosterol (~−27.3 mV for plain and ~−25.0 mV for extract-loaded samples). Bhattacharya et al. [60] and Ricci et al. [61] have reported that sterols caused the change in the phospholipid order, and the thickness of the membrane, influencing the total charge. Hence, the addition of sterol can induce the hydrophobic stabilization of the liposomal bilayer [60]. Additionally, there were statistically significant differences in zeta potential between empty and extract-loaded liposomes. Namely, the extract loading caused the decrease in the zeta potential absolute values, which agrees with the study of red bryony and horned poppy extracts’ encapsulation within the liposomes [62]. This reduction in the absolute value indicates that the Serpylli herba extract partially neutralized the lipid surface charge [63]. The literature suggests that polyphenols and organic acids in the extracts interact with the lipid bilayer, altering surface charge and thus reducing electrostatic repulsion [62]. The zeta potential values below −25 mV in most cases indicate sufficient electrostatic repulsion to maintain colloidal stability [64]. UV-irradiated liposomes with 20 mol% of ergosterol (plain and extract-loaded) and all lyophilized liposomes retained or even increased zeta potential (absolute values). It suggests ergosterol’s protective effect against surface charge alterations under stress. However, in the case of liposomes containing a lower amount of ergosterol (10 mol%), UV treatment causes a decrease in the absolute value of the zeta potential. It occurs probably due to insufficient concentration of ergosterol to protect the lipid bilayer membrane from the UV-induced oxidation [65].

The mobility of liposomal particles, i.e., their migration rate in an electric field, is a direct reflection of their surface charge and hydrodynamic properties. It is routinely measured via electrophoretic light scattering techniques to assess electrokinetic behavior separate from zeta potential determination. The data on the mobility of developed liposomes are presented in Table 1. Non-treated plain liposomes containing 10 mol% of ergosterol had mobility of ~−2.19 µm·cm/V·s, whereas the sample containing 20 mol% of ergosterol showed mobility of ~−1.89 µm·cm/V·s. Liposomes with extract and 10 mol% of ergosterol exhibited significantly lower mobility (~−1.57 µm·cm/V·s). Liposomes with extract and 20 mol% of ergosterol had mobility of ~−1.60 µm·cm/V·s. Namely, Chen [66] demonstrated that the mobility of liposomes is a function of particle size, zeta potential, and bilayer composition. Therefore, the obtained differences between various liposome populations were expected. Particles with lower charge correspondingly had lower mobility, which was also the case with Serpylli herba extract-loaded ergosterol–phospholipid liposomes. Additionally, some bilayer membranes are fluid, flexible, and deformable, while others are rigid, which depend on lipid composition and encapsulated molecules. In our study, plain liposomes with a lower level of ergosterol (10 mol%) showed statistically significantly higher mobility compared to the 20 mol% ergosterol sample. This can be explained by the fact that lower ergosterol content may lead to less rigid and more fluid bilayer structures (the ability to deform). It can alter the distribution of surface charges, and enhance the movement of liposomes in an electric field [30,67]. Since liposomal mobility is influenced by mechanical rigidity and membrane deformability [68], the observed lower mobility of developed liposomes with extract suggests increased stiffness compared to unloaded ones. This increased rigidity may be attributed to the adsorption of flavonoid compounds from the Serpylli herba extract onto the liposomal surface, which can hinder particle movement [69]. The mobility of UV-irradiated plain liposomes with 10 mol% of ergosterol was significantly lower in comparison to the non-treated sample. In other formulations, UV exposure caused an increase in mobility values, suggesting the protective effects of ergosterol and Serpylli herba extract constituents. This reduction in the plain 10 mol% ergosterol formulation could be attributed to UV-induced oxidative changes in the lipid bilayer, potentially leading to partial degradation or crosslinking of phospholipids. This can reduce surface charge availability or rearrange surface groups in a less electrokinetically favorable manner [70]. Conversely, in extract-loaded or higher ergosterol formulations, UV exposure resulted in increased mobility magnitude, suggesting enhanced exposure of anionic surface components. This behavior may stem from UV-triggered structural reorganization of the bilayer in the presence of polyphenols or sterols, both of which can elevate the net surface charge or improve charge accessibility [71]. Polyphenolic constituents may also undergo photochemical changes, contributing to surface charge alteration or modifying the local dielectric environment near the liposome surface, thus increasing mobility [72]. Mobility also showed an increase in magnitude in lyophilized samples in a range from ~−2.02 µm·cm/V·s to ~−2.34 µm·cm/V·s. Nevertheless, in the case of plain liposomes with 10 mol% ergosterol, there was no statistically significant difference. This trend suggests enhanced surface charge expression or rearrangement of surface-bound components under stress conditions, particularly lyophilization, which is known to affect vesicle organization and electrokinetic behavior [73]. The increased mobility magnitude following lyophilization may also reflect partial dehydration-induced rearrangement of the lipid bilayer and improved exposure of negatively charged headgroups, particularly in extract-loaded systems.

### 3.3. Density, Surface Tension, and Viscosity of the Non-Treated and UV-Irradiated Liposomes

To evaluate the influence of formulation composition and UV irradiation on the physical behavior of liposomal systems, the density, surface tension, and viscosity of both unloaded and Serpylli herba extract-loaded liposomes were measured (Table 2). Since the lyophilization process was applied to obtain a dried and longer-term product that is not susceptible to microbial contamination, the mentioned variables (as important parameters for liquid formulations) were not examined in the case of lyophilized liposomes. 95% confidence intervals related to the analyzed data are shown in Table S2.

The density of non-treated and UV-irradiated liposomes enriched with ergosterol (in the absence and presence of Serpylli herba extract) is shown in Table 2. Across all formulations, density values ranged narrowly between 0.996 and 1.001 g/mL, with no statistically significant differences between samples. These findings suggest that ergosterol incorporation, whether alone or in combination with extract, did not markedly alter the bulk density of liposomal dispersions. Ergosterol, as mentioned above, is known to integrate into phospholipid bilayers and modulate membrane properties such as fluidity, packing order, microviscosity, and permeability. However, in terms of macroscopic density, its impact appears negligible at 10–20 mol% inclusion levels. It is probably due to its molecular weight and spatial contribution being comparable to native phospholipids. Similarly, the incorporation of Serpylli herba extract, rich in polyphenolic constituents, did not significantly affect the density of the liposomal formulations. Because liposomes contain relatively small amounts of extract compared with lipids and water, and density reflects bulk mass, the extract’s contribution is too minor to noticeably affect overall density. UV irradiation also did not produce notable shifts in density, suggesting that the structural integrity and dispersion stability of the liposomal suspensions were maintained post irradiation. UV exposure can cause lipid peroxidation or alter bilayer packing [65,74], but these effects typically require higher energy or longer exposure to noticeably affect bulk properties like density. The minimal variation observed here further confirms the robustness of the liposomal architecture, particularly when stabilized by sterol components such as ergosterol, which can enhance bilayer cohesion and resilience to oxidative stress [30,75]. This physicochemical stability is a valuable attribute for pharmaceutical or nutraceutical delivery systems, particularly when environmental stressors (e.g., UV light) are unavoidable during processing or storage.

Surface tension is a critical parameter influencing liposome stability, dispersion behavior, and interaction with biological interfaces [47]. The surface tension values of ergosterol-containing liposomes, both plain and extract-loaded, showed minimal variation, ranging from ~24.9 mN/m to ~26.9 mN/m across all samples (Table 2). Statistical analysis indicated no significant differences between formulations, confirming that neither ergosterol incorporation nor the presence of Serpyli herba extract significantly altered the lipid-water interfacial energy. This aligns with prior findings where sterol molecules, such as cholesterol or ergosterol, primarily modulate bilayer fluidity and packing but exert a limited effect on the macroscopic surface tension of vesicular dispersions [76,77]. The surface tension values remaining close to those of pure phospholipid systems indicate preserved bilayer integrity and the absence of surface-active degradation products that could lower surface tension [78]. Consistent surface tension also suggests stable vesicle morphology and colloidal behavior, essential for predictable liposomal delivery performance. In addition, no significant differences were observed between non-treated and UV-irradiated samples, highlighting the resilience of the liposomal surface properties to mild oxidative stress induced by UV exposure [79].

Viscosity plays a crucial role in determining stability, injectability, and release profiles of liposomal formulations. The viscosity values of the non-treated liposomal formulations varied significantly, ranging from ~15.0 mPa·s to ~22.3 mPa·s (Table 2), with a clear pattern related to ergosterol content and extract loading. Both non-treated plain liposome populations exhibited significantly lower viscosities (15.0–15.7 mPa·s). Non-treated formulation with 20 mol% ergosterol and loaded with extract possessed the highest viscosity, reflecting the combined effect of increased sterol-induced bilayer rigidity and extract-related intermolecular interactions. The statistically significant increases in viscosity upon extract loading (e.g., to ~21.8 mPa·s at 10 mol% of ergosterol) underscore the importance of extract–lipid interactions in modulating liposomal rheology. This suggests that ergosterol strengthens bilayer packing, while extract compounds such as polyphenols can further enhance vesicle–vesicle interactions or bilayer hydration, cumulatively increasing viscosity [30,71,72,75]. These findings have important implications for the design of liposomal formulations, where viscosity affects processing, stability, and delivery characteristics. The elevated viscosity observed in the presence of the extract supports improved membrane integrity but may require optimization for applications demanding lower flow resistance. Differences between non-treated and UV-irradiated formulations suggest that environmental and processing conditions affect viscosity, with lower values (~10.3–16.8 mPa·s) likely resulting from membrane relaxation, slight fluidization, or structural rearrangements [80,81].

Although steroidal compounds, such as ergosterol, are known to influence liposomal bilayer microviscosity, direct measurements of microviscosity were not performed in this study. Future investigations could focus on assessing the impact of ergosterol on membrane microviscosity and related biophysical properties to further elucidate its stabilizing role.

### 3.4. The Storage Stability of Non-Treated Liposomal Vesicles

**Figure 1:**
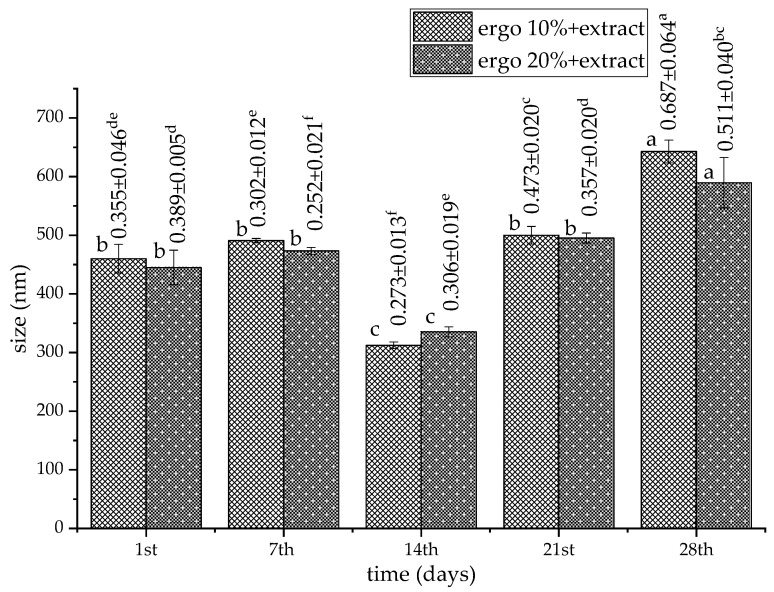
Particle size—bars and polydispersity index—numbers above bars of Serpylli herba extract-loaded liposomes with 10 mol% or 20 mol% of ergosterol (non-treated samples) for 28 days of storage at 4 °C; ergo, ergosterol; different letters showed statistically significant differences (*p* < 0.05; *n* = 3; analysis of variance, Duncan’s post hoc test).

**Figure 2:**
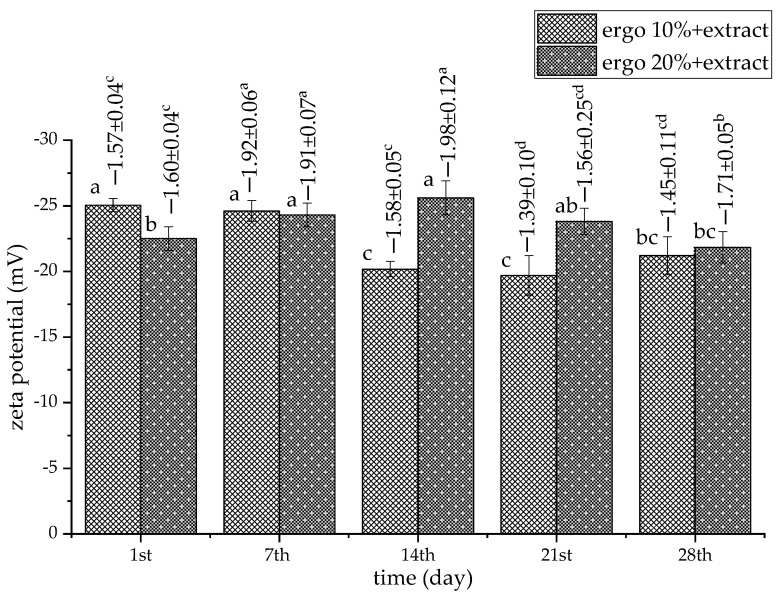
Zeta potential—bars and mobility—numbers above bars (µm·cm/V·s) of Serpylli herba extract-loaded liposomes with 10 mol% or 20 mol% of ergosterol (non-treated samples) for 28 days of storage at 4 °C; ergo, ergosterol; different letters showed statistically significant differences (*p* < 0.05; *n* = 3; analysis of variance, Duncan’s post hoc test).

To examine the storage stability of phospholipid-ergosterol liposomes, particle size, PDI, zeta potential, and mobility were measured for 28 days. The results related to the Serpylli herba extract-loaded liposomes (non-treated sample) are presented in Figure 1 and Figure 2. The data on non-treated plain liposomes are shown in the Supplementary Materials (Figure S1).

The particle sizes of all liposomal samples significantly changed (except in the case of plain liposomes containing 10 mol% of ergosterol) during 28 days of refrigeration storage (Figure 1 and Figure S1A). The size of empty liposomes during the 28-day stability study varied between ~604 nm and ~640 nm (with 10 mol% of ergosterol) and between ~596 nm and ~524 nm (with 20 mol% of ergosterol). The size of liposomes with extract ranged from ~460 nm to ~642 nm (with 10 mol% of ergosterol) and from ~445 nm to ~589 nm (with 20 mol% of ergosterol). A notable size drop on the 7th and 14th days, for the plain 20 mol% ergosterol liposomes (~524 nm and ~312 nm, respectively), suggests possible rearrangement and compaction of the bilayer into more ordered particles [28]. It is plausible that a reduction in liposome size during storage reflects bilayer rearrangement into a more ordered and compact vesicle. Nevertheless, in the case of both types of extract-loaded liposomes, there was no statistically significant difference between the diameters measured on the 1st and 21st days. It is consistent with observations of Yanagihara et al. [82] that cholesterol-modified liposomes suppressed the increase in size for 21 days, likely due to enhanced lipid packing. However, both formulations showed a temporary size decrease on the 7th day (300–350 nm). By the 28th day, they exhibited significant enlargement, reaching ~642.7 nm (for 10 mol% sample) and ~589 nm (for 20 mol% sample), consistent with progressive aggregation or bilayer destabilization over time [83].

PDI in all empty liposomes varied from ~0.235 to ~0.562 (numbers above bars in Figure S1A), while PDI in all extract-loaded liposomes fluctuated from ~0.252 to ~0.687 (numbers above bars in Figure 1). The plain 10 mol% and 20 mol% ergosterol liposomes showed narrow size distribution on the 1st day (~0.235 and ~0.276, respectively), which increased to ~0.562 and ~0.467, respectively, on the 28th day. The 10 mol% ergosterol formulation with extract started with moderate heterogeneity (~0.355), which dropped to ~0.273 on the 14th day, indicating improved homogeneity potentially linked to membrane reorganization. However, by the 28th day, PDI rose to ~0.687, signifying heterogeneous aggregation or fusion (as in the case of empty parallels). In comparison, 20 mol% ergosterol liposomes with extract started with a PDI value of ~0.389 and displayed lower PDI at early time points (e.g., ~0.252 and ~0.306 on the 7th and 14th days, respectively). PDI value also increased by the 28th day (~0.511), but to a lesser extent than the 10 mol% formulations, similar to the plain samples. This suggests that higher ergosterol content contributes to improved vesicle stability, likely due to rigid bilayer formation and enhanced steric hindrance [30,40,84]. Since the Serpylli herba polyphenol-loaded, ergosterol-enriched liposomes were prepared using the proliposome method, slightly higher PDI values were expected. PDI values below 0.3 are generally considered ideal for uniform particle size. Nonetheless, although this technological approach results in a moderately heterogeneous liposomal dispersion system, it also provides several advantages. These advantages include ease of handling and scalability, reproducible liposome formation upon hydration, and flexibility in composition. While the PDI values observed were slightly above 0.3 in some formulations, this is consistent with the inherent variability of liposomes derived from proliposomes and does not compromise encapsulation efficiency or structural integrity. The proliposome method allows the incorporation of ergosterol and polyphenols into liposomes efficiently, supporting the robustness of the formulation for potential applications. Optimization of particle uniformity could be addressed in future studies.

The surface charge of the liposomes, represented by zeta potential, varied in all samples, but the trend depended on the composition of the liposomal membrane and the absence or presence of the extract. In addition, it remained consistently negative throughout the storage period (Figure 2 and Figure S1B). In the empty liposomes with 10 mol% of ergosterol, zeta potential (absolute value) decreased until the 7th day, from ~−27.3 mV to ~−22.7 mV. After that, zeta potential (absolute value) increased up to ~−26.4 mV (on the 28th day) (Figure S1B). In the empty liposomes containing 20 mol% of ergosterol, zeta potential decreased until the 28th day, from ~−24.3 mV to ~−22.2 mV. The zeta potential of extract-loaded liposomes with 10 mol% ergosterol ranged from ~−25.0 mV (1st day) to ~−21.2 mV (28th day). The 20 mol% parallel showed a less negative value on the 1st day (~−22.5 mV), without changing until the 28th day (~−22.2 mV) (Figure 2). Zeta potential around or above −20 mV is considered moderately stable, though not strongly resistant to aggregation [40]. According to the literature data, ergosterol can maintain electrostatic stability, possibly due to enhanced membrane order and more persistent surface charge distribution [84,85].

Mobility values followed similar trends to zeta potential, showing negative values for all developed liposomal systems during storage. In plain 10 mol% ergosterol liposomes, mobility reached values of ~−2.19 µm·cm/V·s (1st day) and ~−2.04 µm·cm/V·s (28th day). The plain 20 mol% ergosterol formulation showed mobility of ~−1.89 µm·cm/V·s (1st day) and ~−1.72 µm·cm/V·s (28th day) (Figure S1B, values above bars). Additionally, the mobility of extract-loaded liposomes was not significantly different on the 1st and 28th days of storage (Figure 2, numbers above bars). Namely, the mobility was between ~−1.57 µm·cm/V·s and ~−1.45 µm·cm/V·s (for 10 mol% ergosterol formulation), and between ~−1.60 µm·cm/V·s and ~−1.71 µm·cm/V·s (for 20 mol% ergosterol formulation). These values indicate adequate electrokinetic stability but also imply a tendency toward fluctuation, possibly due to dynamic rearrangement of membrane components. Importantly, no drastic mobility drop was observed, confirming persistent surface charge, even during particle growth [86].

### 3.5. The Storage Stability of UV-Irradiated Liposomes

**Figure 3:**
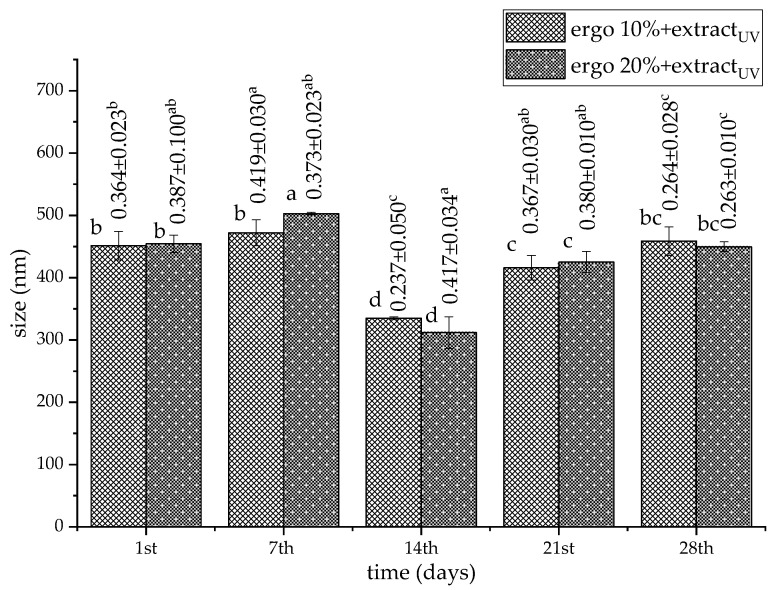
Particle size—bars and polydispersity index—numbers above bars of Serpylli herba extract-loaded liposomes with 10 mol% or 20 mol% of ergosterol (UV-treated samples) for 28 days of storage at 4 °C; ergo, ergosterol; different letters showed statistically significant differences (*p* < 0.05; *n* = 3; analysis of variance, Duncan’s post hoc test).

**Figure 4:**
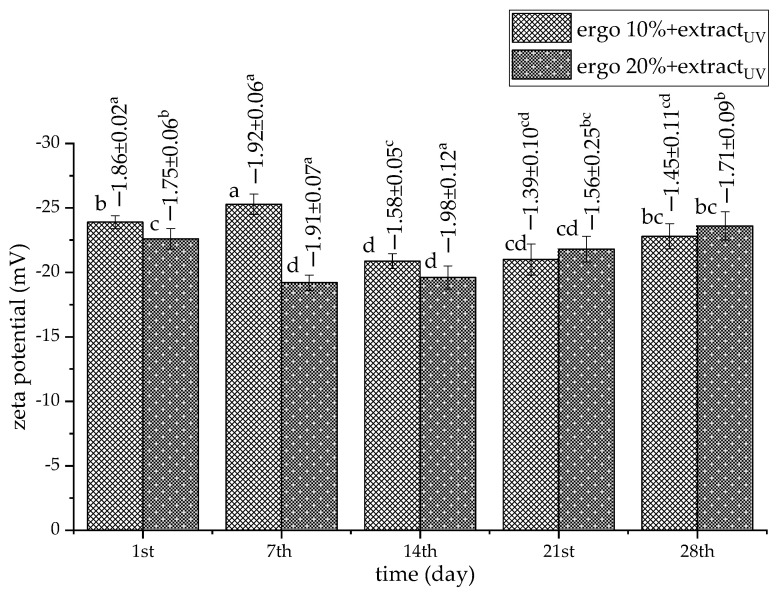
Zeta potential—bars and mobility—numbers above bars (µm·cm/V·s) of Serpylli herba extract-loaded liposomes with 10 mol% or 20 mol% of ergosterol (UV-treated samples) for 28 days of storage at 4 °C; ergo, ergosterol; different letters showed statistically significant differences (*p* < 0.05; *n* = 3; analysis of variance, Duncan’s post hoc test).

The stability of UV-irradiated liposome size during storage is a critical parameter that influences their physicochemical behavior, biological performance, and shelf-life. Storage stability of the UV-irradiated liposomes is shown in Figure 3 and Figure 4 (for extract-loaded formulations) and Figure S2 (for plain liposomes).

The diameters of vesicles in all UV-treated liposomal populations significantly changed (except for unloaded liposomes with 10 mol% of ergosterol) during 28 days of storage (Figure 3 and Figure S2A). UV-irradiated extract-loaded liposomes formulated with 10 mol% of ergosterol showed size fluctuations throughout the storage period, with an initial average size of ~451 nm, peaking at ~472 nm by the 7th day. It is possibly due to osmotic swelling or minor vesicle fusion during early equilibration [87,88]. Further, it decreased substantially to ~334 nm by the 14th day, and then rose again to ~458 nm on the 28th day (Figure 3). UV-irradiated extract-loaded liposomes with 20 mol% of ergosterol did not demonstrate consistent behavior as well. Their size values ranged from ~454 nm to ~449 nm, and a particularly sharp increase was observed on the 7th day (~503 nm) and a marked drop on the 14th day (~312 nm) (Figure 3). The subsequent recovery to 425–449 nm by the 21st and 28th days suggests partial restructuring or re-stabilization over time. The fluctuations in liposome size may be attributed to vesicle fusion, aggregation, or bilayer rearrangement processes during storage, which are typical in liposomal systems not sufficiently stabilized [83,89]. The transient reduction in size observed on the 14th day could result from osmotic stress or reorganization of the lipid bilayer, potentially induced by storage-induced dehydration/rehydration cycles or temperature effects [90]. Higher ergosterol content may enhance bilayer rigidity and reduce permeability, contributing to improved vesicle stability upon UV irradiation. Indeed, several studies have shown that increased sterol content in liposomes leads to tighter lipid packing and reduced fusion or leakage during storage [30,31,40,46]. However, excessive sterol can also lead to phase separation or altered curvature, explaining the transient instability at the 7th day in the 20 mol% ergosterol liposomes.

PDI values of all UV-irradiated empty liposomes varied in a wide range, from ~0.251 to ~0.612 (numbers above bars in Figure S2A), as in the case of non-treated counterparts. Specifically, the plain 10 mol% and 20 mol% ergosterol liposomes after UV irradiation showed size distribution from ~0.251 and ~0.255, respectively (on the 1st day) to ~0.612 and ~0.521, respectively (on the 28th day). As shown in Figure 3 (the numbers above bars), the PDI of UV-treated liposomes with 10 mol% ergosterol and extract began at ~0.364, peaking at ~0.419 on the 7th day, and decreasing steadily to ~0.264 by the 28th day. This trend indicates an initial increase in size heterogeneity, likely due to vesicle fusion or aggregation during early storage, followed by progressive stabilization of the liposomal population. Notably, the lowest PDI was recorded on the 14th day, potentially reflecting a transient reorganization of vesicles into a more uniform population. UV-irradiated liposomes with 20 mol% ergosterol and extract showed a more consistent PDI, fluctuating between ~0.263 and ~0.387 over 28 days, without the notable drop observed on the 14th day. Despite a higher PDI value on the 14th day (~0.417), values returned to low levels by the 28th day, suggesting that higher ergosterol content may contribute to restoring and maintaining vesicle uniformity after destabilizing events. The initial PDI increase in both formulations may result from thermodynamic instability of freshly prepared liposomes under UV treatment, where residual solvent, vesicle fusion, or incomplete equilibration broadens the size distribution. The slightly better performance of 20 mol% ergosterol liposomes in maintaining a consistent PDI suggests that this higher content may offer superior steric or interfacial stabilization. Both extract-loaded formulations reached PDI values <0.3 by the 28th day, meeting commonly accepted criteria for liposomal uniformity.

In this study, UV-irradiated plain liposomes showed different trends in zeta potential values during storage in comparison to non-treated counterparts. Namely, the absolute values of the zeta potential increased with time and achieved the values of ~−30 mV (Figure S2B). It is probably due to the reorganization of phospholipids in the liposomal bilayer upon UV irradiation. UV-treated liposomes with 10 mol% ergosterol and extract showed relatively stable negative zeta potential values, ranging from ~−25.28 mV (7th day) to ~−20.87 mV (14th day), returning to ~−22.8 mV (28th day) (Figure 4). This moderate and consistent negative surface charge suggests a stable colloidal system with limited aggregation tendencies. The initial increase in negativity on the 7th day may indicate surface reorganization or enhanced exposure of anionic groups, followed by gradual equilibration during storage. UV-irradiated liposomes with 20 mol% ergosterol and extracts exhibited a similar trend, but with more variation in zeta potential values. Namely, values ranged from ~−22.6 mV on the 1st day to a minimum of ~−19.2 mV on the 7th day, and stabilized again by the 28th day at ~−23.6 mV. The temporary reduction in surface charge on the 7th day suggests partial screening or reorientation of lipid headgroups, potentially influenced by ergosterol’s effect on membrane packing and surface exposure. The slightly less negative zeta potential values in the 20 mol% ergosterol formulation with extract, particularly on the 7th and 14th days, may reflect tighter bilayer packing that limits the availability of charged phospholipid headgroups at the surface. Despite these fluctuations, both UV-treated extract-loaded liposomal formulations had maintained zeta potential values between ~−19.2 mV and ~−25.3 mV, indicating moderate electrostatic stability throughout the storage period. This level of surface charge is typically sufficient to prevent extensive aggregation in phospholipid vesicles, particularly when combined with steric stabilization provided by bilayer rigidity or associated macromolecules. The observed stabilization by the 28th day, particularly in the 20 mol% ergosterol system (~−23.6 mV), suggests that the bilayers reorganized into more thermodynamically stable conformations over time. These findings support the hypothesis that ergosterol plays a dual role, both modulating bilayer order and indirectly affecting colloidal stability through changes in surface potential.

The absolute values of mobility of UV-treated unloaded liposomes increased with time and achieved the values of ~−2.4 µm·cm/V·s (Figure S2B, numbers above bars). UV-treated liposomes with 10 mol% ergosterol and extract showed changes in the mobility values, ranging from ~−1.86 µm·cm/V·s on the 1st day to ~−1.45 µm·cm/V·s on the 28th day (Figure 4, numbers above bars). A slight drop in negativity was observed on the 14th day (~−1.58 µm·cm/V·s) and the 21st day (~−1.39 µm·cm/V·s). It possibly indicates temporary alterations in the surface charge environment, such as lipid rearrangement or vesicle fusion. However, the recovery to a more negative value on the 28th day suggests re-stabilization of the bilayer and charge distribution over time. UV-irradiated liposomes with 20 mol% ergosterol and extract also exhibited dynamic changes. Their mobility ranged from ~−1.75 µm·cm/V·s (1st day) to a maximum of ~−1.98 µm·cm/V·s on the 14th day, indicating stronger surface charge at mid-storage. This pronounced increase in negative mobility might be attributed to tightened bilayer packing and increased exposure of anionic phospholipid groups, facilitated by the higher ergosterol content, as previously mentioned. Nevertheless, the values returned to ~−1.71 µm·cm/V·s by the 28th day, suggesting a gradual surface charge equilibration. The stronger negative mobility observed in the 20 mol% ergosterol extract-loaded liposomes between the 7th and 14th days corresponds with potential electrostatic reorganization at the vesicle surface or reorientation of sterol-phospholipid interactions. The 20 mol% ergosterol system exhibited slightly more variability, which might reflect higher sensitivity to bilayer restructuring events or heterogeneity in sterol distribution within the membrane. Overall, both liposomal formulations maintained negative electrophoretic mobility throughout the 28-day storage, confirming the presence of stable anionic surfaces.

The improved stability observed in ergosterol-containing liposomes may be attributed to the unique chemical structure of ergosterol, which contains additional unsaturated bonds that influence lipid packing and reduce susceptibility to oxidative degradation. Unlike cholesterol, which is highly effective in decreasing membrane fluidity but can lead to rigid bilayers under stress, ergosterol confers both stabilizing and protective effects without compromising efficiency of the encapsulation [30,31]. In addition, its fungal origin provides an advantage over animal-derived cholesterol, reinforcing the novelty of our approach and demonstrating that ergosterol represents a valuable alternative sterol for designing robust and sustainable liposomal systems.

### 3.6. The Storage Stability of the Lyophilized Liposomes

**Figure 5:**
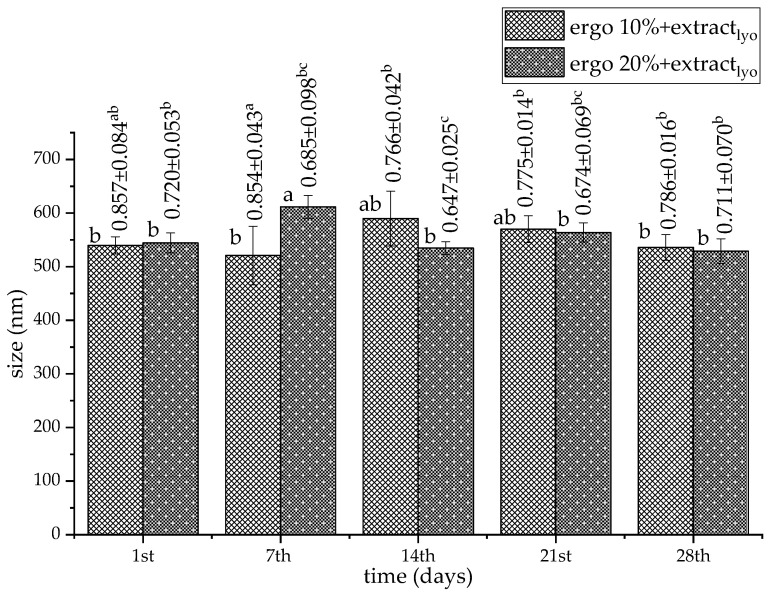
Particle size—bars and polydispersity index—numbers above bars of Serpylli herba extract-loaded liposomes with 10 mol% or 20 mol% of ergosterol (lyophilized samples) for 28 days of storage at 4 °C; ergo, ergosterol; different letters showed statistically significant differences (*p* < 0.05; *n* = 3; analysis of variance, Duncan’s post hoc test).

**Figure 6:**
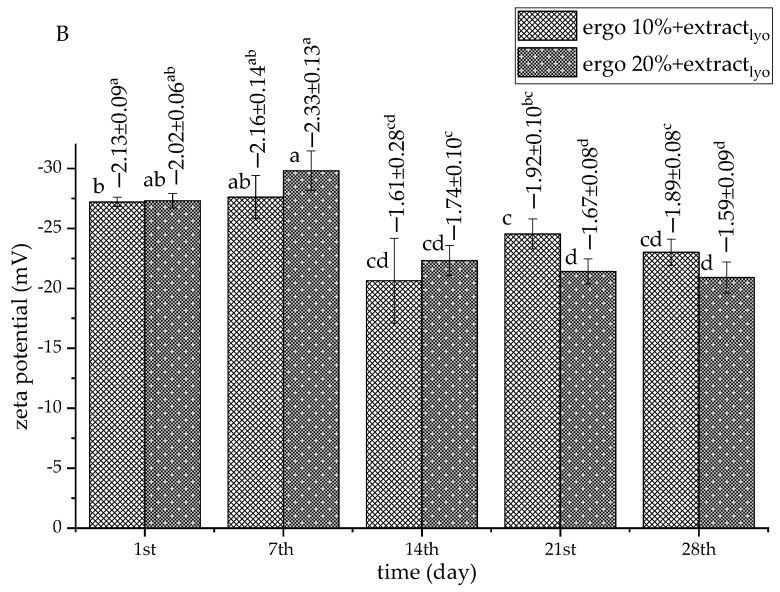
Zeta potential—bars and mobility—numbers above bars (µm·cm/V·s) of Serpylli herba extract-loaded liposomes with 10 mol% or 20 mol% of ergosterol (lyophilized samples) for 28 days of storage at 4 °C; ergo, ergosterol; different letters showed statistically significant differences (*p* < 0.05; *n* = 3; analysis of variance, Duncan’s post hoc test).

Lyophilization is a widely applied technique for improving the long-term stability of liposomal formulations, with significant impact on liposome structure, homogeneity, and surface characteristics, particularly in the absence of an effective cryoprotectant. Storage stability of the lyophilized liposomes with extract for 28 days is presented in Figure 5 and Figure 6, while data related to lyophilized plain liposomes are presented in Figure S3.

The diameters of all lyophilized liposomes (unloaded and extract-loaded) exhibited slight fluctuations across the storage period (Figure 5 and Figure S3A). Lyophilized plain liposomes with 10 mol% and 20 mol% of ergosterol showed an initial size of ~637.5 nm and ~644.3 nm, respectively. In addition, vesicle sizes were ~630.5 nm and ~646.1 nm, respectively, by the 28th day (Figure S3A). Lyophilized extract-loaded liposomes with 10 mol% of ergosterol showed an initial size of ~539.7 nm and ~536.5 nm by the 28th day (Figure 5). Lyophilized 20 mol% ergosterol liposomes with extract demonstrated greater initial enlargement (~544.3 nm) and a notable increase on the 7th day (~611.7 nm), followed by a decline to ~529.0 nm by the 28th day (Figure 5). These results indicate that lyophilization can transiently destabilize vesicles, possibly due to phase separation or incomplete reconstitution after freeze-drying. By the 28th day, the return to near-initial sizes, in both formulations, reflects gradual rehydration and membrane reassembly, with ergosterol likely supporting bilayer integrity. PDI values for lyophilized 10 mol% ergosterol liposomes (plain and extract-loaded) remained relatively high throughout storage. It starts at ~0.857 and ends at ~0.786 (for the extract-loaded sample, Figure 5, numbers above bars), and with an initial value of ~0.846 and ending at ~0.715 (for the plain sample, Figure S3A, values above bars), indicating broad size distributions. Lyophilized plain liposomes containing 20 mol% of ergosterol also showed a very high value of PDI during storage (0.787–0.720). Meanwhile, lyophilized 20 mol% ergosterol extract-loaded liposomes consistently displayed high PDI values (from ~0.720 to ~0.711), suggesting a more uniform vesicle population post reconstitution. The consistently better PDI performance of the 20 mol% ergosterol group indicates that higher sterol content contributes to improved bilayer resilience and homogeneity. It is likely due to enhanced membrane rigidity and protection against fusion or collapse during freeze-drying [30,31,46]. This emphasizes the protective role of sterols in preserving liposomal morphology during lyophilization. The high PDI values observed in lyophilized formulations are primarily attributable to the challenges associated with freeze-drying proliposome-derived liposomes containing polyphenol-rich extracts. While the present study focused on the characterization of the prepared formulations, further optimization could improve particle size uniformity. Potential strategies can include optimization of freeze-drying parameters via adjusting freezing rate, primary drying temperature, and secondary drying conditions, consequently reducing aggregation and improving rehydration. Additional strategies can be the use of cryoprotectant sugars or polyols, such as trehalose or sucrose, to stabilize the lipid bilayer during lyophilization and reduce PDI upon rehydration. The last strategy may include optimization of the hydration protocols in terms of controlled rehydration of proliposomes to minimize liposome fusion and polydispersity. Implementation of these strategies in future studies could enhance particle uniformity while maintaining the advantages of the proliposome method, including stability, ease of handling, and reproducible liposome formation.

Zeta potential values across both lyophilized formulations were negative and indicated moderate electrostatic stability. Lyophilized plain 10 mol% ergosterol liposomes showed zeta potential values in a range of ~−26.8 mV (1st day) and ~−24.0 mV (28th day). The zeta potential of lyophilized unloaded 20 mol% ergosterol liposomes varied from ~−28.9 mV to ~−20.9 mV (Figure S3B). For lyophilized 10 mol% ergosterol liposomes with extract, zeta potential ranged from ~−27.6 mV to ~−23.0 mV. The zeta potential of lyophilized 20 mol% ergosterol extract-loaded liposomes fluctuated between ~−27.3 mV and ~−25.2 mV (Figure 6). Both lyophilized extract-loaded liposome groups showed a temporary decrease in surface charge on the 14th day, likely due to interfacial rearrangements and hydration changes during freeze-drying, with values returning by the 28th day. Lyophilized unloaded 10 mol% ergosterol liposomes possessed mobility of ~−2.10 µm·cm/V·s (1st day) and ~−1.82 µm·cm/V·s (28th day). The mobility of lyophilized plain 20 mol% ergosterol liposomes fluctuated from ~−2.34 µm·cm/V·s to ~−1.98 µm·cm/V·s (Figure S3B, numbers above bars). For the lyophilized 10 mol% ergosterol sample with extract, mobility ranged from ~−2.13 µm·cm/V·s to ~−1.89 µm·cm/V·s. For the 20 mol% ergosterol sample with extract, mobility values varied from ~−2.02 µm·cm/V·s to ~−1.59 µm·cm/V·s (Figure 6, the numbers above bars). The more negative values in the 10 mol% ergosterol extract-loaded liposome group after a 28-day storage could reflect greater surface exposure of negatively charged lipids. On the other hand, the 20 mol% ergosterol extract-loaded liposomes showed lower magnitude mobility on the 28th day, possibly due to denser sterol-lipid packing reducing surface charge accessibility, as previously mentioned.

Ergosterol differs from cholesterol by the presence of additional double bonds in the B-ring (C7–C8) and in the side chain (C22–C23), which introduce slight conformational rigidity while maintaining a degree of membrane fluidity. These unsaturations enhance lipid tail packing and bilayer order, reducing the propensity for membrane defects and leakage. Compared with cholesterol, which primarily stabilizes membranes through rigid planar stacking, ergosterol’s unsaturation allows for a balance between rigidity and flexibility, improving resistance to oxidative stress and thermal or mechanical perturbation. Molecular dynamics studies have shown that ergosterol-containing membranes exhibit increased thickness, higher order parameters, and reduced lateral diffusion, all of which contribute to the improved physical and oxidative stability observed in our liposomal formulations [33,91]. These features likely underpin the enhanced retention of polyphenolic bioactives and preservation of vesicle integrity during lyophilization and storage.

Although no cryoprotectants were used in the present study, the results demonstrate that ergosterol alone provides substantial stabilization of liposomal vesicles during lyophilization and storage. This finding highlights the intrinsic protective capacity of ergosterol within the bilayer, preserving particle size and zeta potential during storage. Future studies may incorporate conventional cryoprotectants to further enhance long-term stability, but the current data confirm the practical potential of ergosterol-based liposomes as robust carriers for pharmaceutical and nutraceutical applications.

### 3.7. NTA and TEM Analyses of the Liposomes

**Figure 7:**
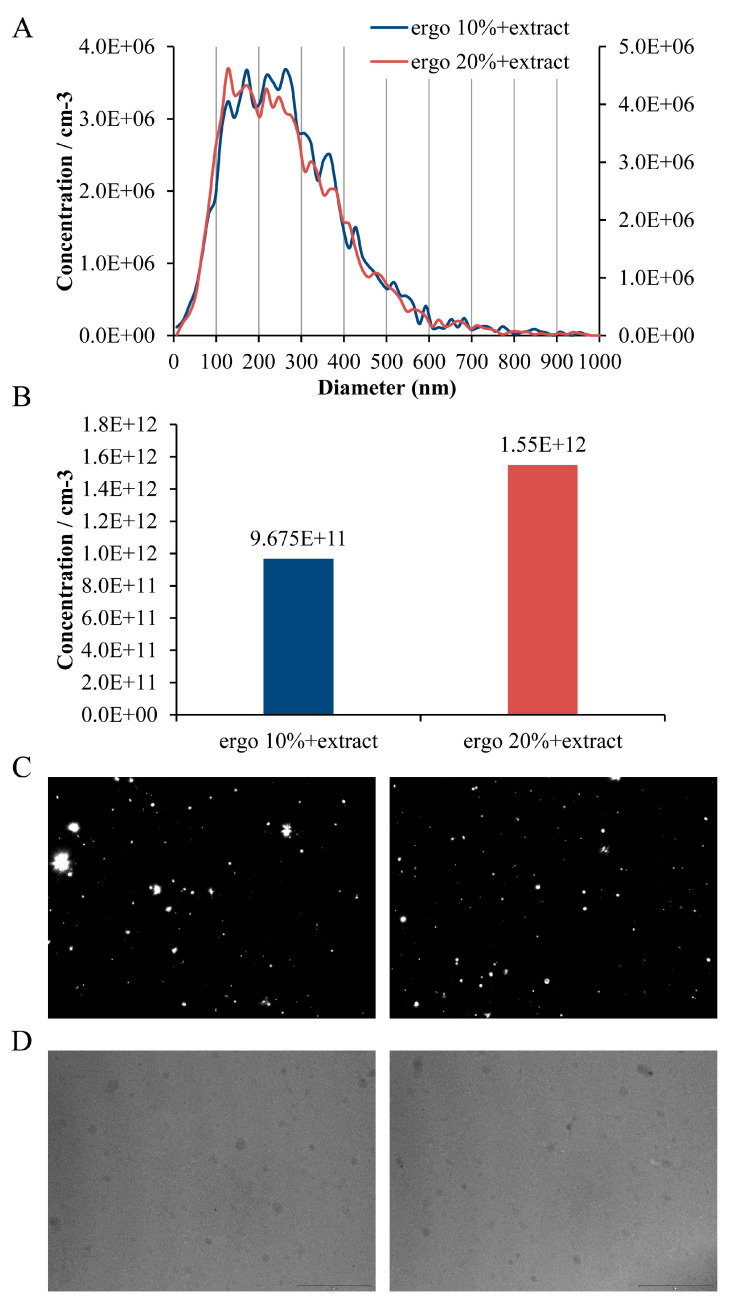
Characterization of Serpylli herba extract-loaded liposomes with 10 mol% or 20 mol% of ergosterol (non-treated samples) using nanoparticle tracking analysis (NTA) and transmission electron microscopy (TEM); (**A**) size distribution, (**B**) particle concentration of liposomes, (**C**) representative NTA video frame captures showing the particles in each preparation, and (**D**) TEM images illustrating the size heterogeneity and morphology of the liposomes (scale bar—2 µm); ergo, ergosterol.

NTA was used to evaluate the particle size distribution and concentration of Serpylli herba extract-loaded liposomes enriched using 10 mol% and 20 mol% of ergosterol. The results are presented in Figure 7A–C.

NTA revealed that both liposome formulations exhibited comparable size heterogeneity. Furthermore, developed liposomes display particle concentrations of 9.67 × 10^11^ particles/mL (for 10 mol% ergosterol liposomes) and 1.55 × 10^12^ particles/mL (for 20 mol% ergosterol liposomes). Although both liposome types were prepared with the same phospholipid and extract concentrations, differences in ergosterol content led to variations in particle number. These results highlight how membrane composition influences vesicle formation and provide insights into potential dose uniformity in delivery applications. Broad size distributions presented here are common in conventional liposomes, particularly when prepared by passive loading techniques such as thin-film hydration or proliposome methods, which often result in vesicle heterogeneity due to variable encapsulation efficiencies and lamellarity. These results indicate that vesicle concentration is modulated by ergosterol inclusion in a concentration-dependent manner, with potential impacts on biological behavior, release kinetics, and formulation stability.

TEM analysis of developed ergosterol-enriched liposomes loaded with Serpylli herba extract revealed well-formed vesicles with preserved structural integrity (Figure 7D). Both samples exhibited size heterogeneity, which agrees with the PCS measurement (Section 3.3), reflecting variations in vesicle diameter and distribution. Together, TEM and PCS analyses provide complementary insights into liposome morphology, size distribution, and stability. Comparison of TEM and PCS data demonstrates that while PCS provides quantitative hydrodynamic size and polydispersity, TEM offers direct visualization of vesicle morphology, confirming the integrity of the liposomal structures. No significant aggregation or deformation was observed, suggesting that ergosterol contributes to membrane rigidity and structural stability. The presence of the Serpylli herba extract may also influence morphology by inserting into or interacting with the lipid bilayer. TEM imaging confirmed that ergosterol-enriched liposomes maintained spherical morphology and intact bilayers after loading with Serpylli herba extract. These results indicate that ergosterol not only stabilizes the bilayer but may also facilitate homogeneous incorporation of plant bioactives without compromising vesicle architecture.

### 3.8. Polyphenol Release from the Liposomes

**Figure 8:**
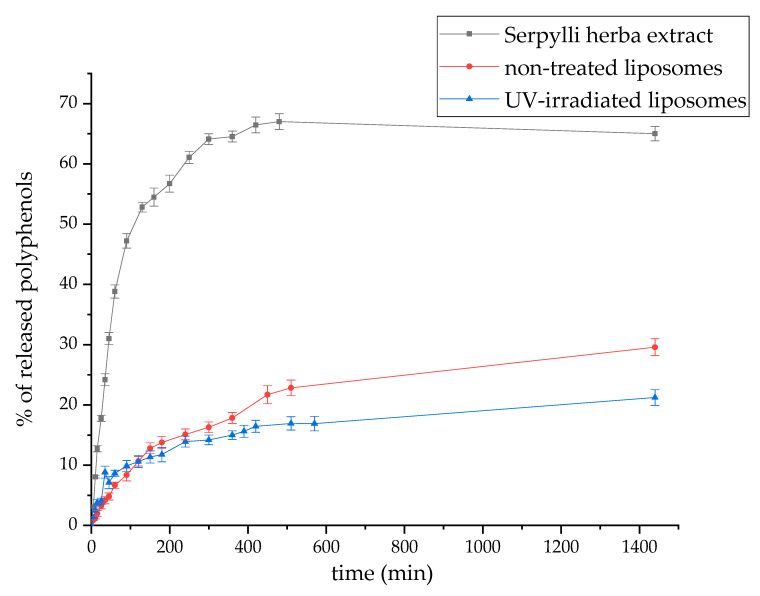
Release profiles of polyphenols from pure Serpylli herba extract and non-treated and UV-irradiated liposomes with extract and 20 mol% of ergosterol, in water, using a Franz diffusion cell.

The release studies of polyphenols were done using a Franz diffusion cell to quantify the mass transfer resistance of the liposomal membrane. The results are shown in Figure 8, where the percentage of released polyphenols is given as a function of time for a period of 24 h in a water medium. The release of polyphenols from 20 mol% ergo liposomes (non-treated and UV-irradiated samples) was compared with the diffusion profile of polyphenols from pure Serpylli herba extract, which was used as a control (with the same extract concentration as the one used for liposomes preparation). Samples with the highest EE, i.e., liposomes with 20 mol% of ergosterol (non-treated and UV-irradiated), were selected to be examined. Due to the significant decrease in EE of polyphenols after lyophilization, lyophilized samples were not considered for the release study.

As presented in Figure 8, diffusion of polyphenols from the pure extract occurred very fast, and the content of polyphenols in the acceptor compartment reached a maximum after about 300 min in water. In the same medium, the release of polyphenols from liposomes was slower, which was expected. After 24 h, ~65.0% of polyphenols were released from pure extract, whereas only ~29.6% and ~21.2% were released from non-treated and UV irradiated liposomes, respectively. According to these results, liposomes can retain polyphenols and thus be used for their prolonged release, which is significant for future applications.

The results obtained in the release studies were analyzed to determine the diffusion coefficient (D) and diffusion resistance (R) derived from the liposomal bilayers in water. The results are presented in Table 3. The coefficient of polyphenol diffusion from pure Serpylli herba extract and extract-loaded liposomes was calculated from the slope of the linear part of the curve defined by plotting $\ln\left(\frac{{C_{d}^{0}-C}_{r}^{0\;}}{C_{d}{-C}_{r}}\right)$ vs. time, t (Figure S4):

(4) $$\ln\left(\frac{{C_{d}^{0}-C}_{r}^{0\;}}{C_{d}{-C}_{r}}\right)=\;D\;\cdot \;\beta \;t\;$$

where C_D_ and C_R_ are concentrations of polyphenols detected in donor and receptor compartments at time t, respectively; C_D_^0^ and C_R_^0^ are concentrations of polyphenols at the beginning of the experiment; D is the diffusion coefficient; β is the geometrical constant typical for the Franz cell geometry and amounted to 2.49 × 10^4^ m^−2^.

R of the liposomal membrane can be calculated based on membrane thickness δ and D using Equation (5):

(5) $$R=\;\frac{\delta }{D}$$

R represents the cumulative resistance of a semipermeable acetate-cellulose membrane and the resistance of a liposomal bilayer. The contribution of the resistance, which is generated by the synthetic membrane, is determined from the diffusion of polyphenols from the pure extract. Then, liposome resistance was determined from a subtraction of the membrane resistance from the overall diffusion resistance.

The diffusion parameters presented in Table 3 demonstrate clear differences between the free Serpylli herba extract and its liposomal formulations. The extract in aqueous solution exhibited the highest diffusion coefficient (5.76 × 10^−9^ m^2^/s) and the lowest diffusion resistance (3.54 × 10^5^ s/m), consistent with the unrestricted mobility of small polyphenolic constituents in solution. In contrast, non-treated liposomes displayed a markedly reduced diffusion coefficient (9.37 × 10^−10^ m^2^/s), accompanied by a higher resistance (2.17 × 10^6^ s/m), reflecting the diffusional barrier imposed by the lipid bilayer. Additionally, this describes the ability of phospholipid membranes to restrict molecular transport by enhancing structural rigidity and reducing permeability [92,93]. Upon UV irradiation, liposomes showed a partial recovery of diffusion mobility (D = 1.81 × 10^−9^ m^2^/s; R = 1.13 × 10^6^ s/m). This trend may be explained by photoinduced perturbations of the bilayer, leading to increased membrane fluidity and a relaxation of the sterol–phospholipid interactions. Namely, in sterol-enriched vesicles, oxidative or photo-oxidative stress triggered partial bilayer disruption and altered permeability [94]. The decrease in diffusion resistance after irradiation suggests that UV exposure compromises membrane compactness, thereby facilitating molecular release. Interestingly, the presence of 20 mol% ergosterol appears to play a dual role. While ergosterol is known to improve bilayer packing and reduce permeability under normal conditions, at higher concentrations or under oxidative stress, sterols may undergo self-oxidation, producing oxysterol species that destabilize the membrane [95]. This could account for the observed increase in diffusivity of UV-treated liposomes, consistent with reports that sterol-rich membranes are more prone to structural reorganization under stress, as previously explained. These findings highlight the protective function of the liposomal bilayer in retarding extract diffusion, while also demonstrating its susceptibility to photodegradation. Compared with the free extract, which diffuses readily, liposomal encapsulation provides a clear barrier to molecular transport. However, UV irradiation partially attenuates this barrier, pointing to the need for stabilization strategies, such as antioxidant co-loading or surface polymeric coatings.

### 3.9. Antioxidant Capacity of the Liposomes

**Figure 9:**
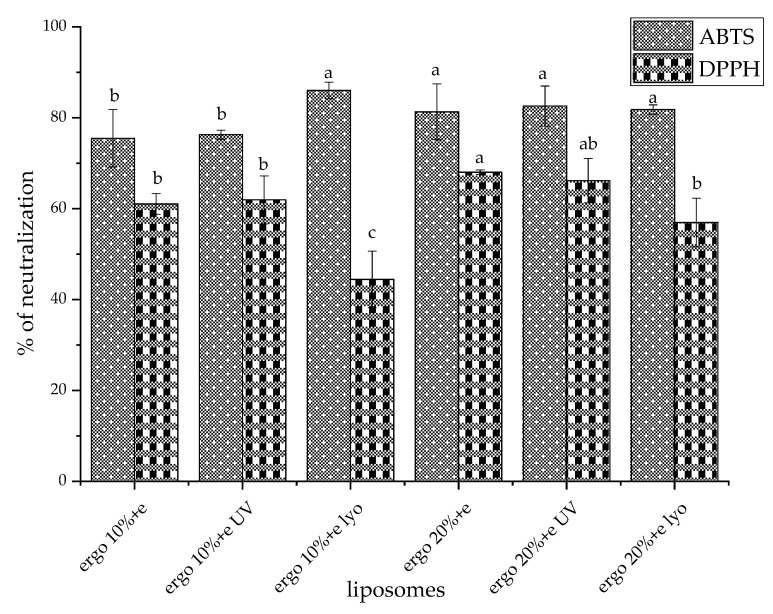
Antioxidant capacity of Serpylli herba extract loaded liposomes (non-treated, UV-irradiated, and lyophilized) obtained in ABTS and DPPH assays; Values with different letters (for each antioxidant test separately) showed statistically significant differences (*p* < 0.05; *n* = 3; analysis of variance, Duncan’s post hoc test); ergo, ergosterol; e, extract.

The antioxidant capacity of Serpylli herba polyphenol-loaded liposomes was systematically evaluated using ABTS, DPPH, and FRAP assays to quantify radical scavenging and ferric ion-reducing potential of developed liposomes. These tests allowed direct comparison of how different liposomal formulations, including various ergosterol contents, retained, enhanced, or decreased antioxidant functionality under UV irradiation and after lyophilization. The results obtained in the above-mentioned assays are presented in Figure 9 (ABTS and DPPH assays) and Table 4 (FRAP assay). 95% confidence intervals related to the analyzed data are shown in Table S3.

The antioxidant capacity of Serpylli herba polyphenol-loaded liposomes, assessed using ABTS, DPPH, and FRAP assays (Figure 9 and Table 4, respectively), demonstrated the retention of significant radical scavenging and reducing activity across all formulations. Extract-loaded liposomes containing 20 mol% ergosterol exhibited higher antioxidant activity in both ABTS and DPPH assays compared to those with 10 mol% ergosterol. For instance, the non-treated ergosterol 20 mol% liposomes with extract showed ABTS scavenging activity of ~81.29%, compared to ~75.48% for the 10 mol% ergosterol counterpart. Similarly, the anti-DPPH activity increased from ~61.02% to ~68.01% with increased ergosterol content. This enhancement may be attributed to the membrane-stabilizing effect of ergosterol, which can influence the encapsulation efficiency (also shown in Section 3.1) and stability of antioxidant compounds (e.g., polyphenols). Ergosterol integrates into the lipid bilayer and can reduce permeability while enhancing the retention of hydrophilic antioxidants in the aqueous core or at the membrane interface [33]. Its presence may also contribute to reducing oxidative degradation by improving membrane rigidity (as previously mentioned), limiting oxygen diffusion into the bilayer.

The encapsulation of the extract in ergosterol-containing liposomes preserved antioxidant potential after UV irradiation and lyophilization (ABTS assay). In the case of anti-DPPH potential, lyophilization caused a drop in the antioxidant activity of developed liposomes. UV-treated liposomes exhibited antioxidant activity comparable to that of non-treated liposomes. For example, UV-treated liposomes with 20 mol% ergosterol and extract neutralized ABTS and DPPH radicals in the amount of ~82.55% and ~66.16%, respectively, marginally higher or like their non-treated counterparts. The fact that UV irradiation did not significantly diminish antioxidant capacity in all formulations can be attributed to UV-induced conformational or phase alterations in the lipid bilayer, potentially improving polyphenol interaction with the aqueous phase or enhancing extract accessibility at the surface [68]. The maintained antioxidant activity suggests that ergosterol’s antioxidant-stabilizing role may mitigate such degradation [37]. A previous study has reported that UV treatment can induce structural changes in liposomal membranes without necessarily degrading encapsulated polyphenolics, particularly when stabilized with sterols like ergosterol [96]. However, it is important to consider that prolonged UV exposure could potentially degrade some polyphenolic components. Lyophilization, while often associated with partial oxidation or leakage, preserved most of the antioxidant activity of developed liposomes with Serpylli herba extract, suggesting good structural integrity and minimal degradation during freeze-drying. Nevertheless, the antioxidant profile of lyophilized samples showed a divergent pattern between ABTS and DPPH assays. Notably, ABTS activity was maintained in both ergosterol formulations post lyophilization (~76.11% for 10 mol% ergosterol and ~82.09% for 20 mol% ergosterol). In contrast, the DPPH inhibition values declined, particularly for the 10 mol% ergosterol sample (~44.42%). Since the drop in the anti-DPPH potential of 20 mol% ergosterol liposomes was lower, this can suggest that higher sterol content can offer better protection of antioxidants during freeze-drying. It is possibly caused by preserving bilayer integrity and minimizing leakage or structural collapse during the sublimation step [97]. This discrepancy may stem from different mechanisms between ABTS and DPPH methods. Namely, the ABTS assay can be performed in both aqueous and lipid phases and can detect both hydrophilic and lipophilic antioxidants, whereas DPPH is more restricted to hydrophobic environments [98]. Furthermore, lyophilization can alter liposomal structure and reorganize extract components, which may affect the location and availability of antioxidants differently in each assay system [53]. The drying process may concentrate antioxidants, leading to increased anti-ABTS values, while partial degradation or reorientation of polyphenol compounds could reduce DPPH inhibition activity [99]. Therefore, the antioxidant potential of Serylli herba extract-loaded liposomes was evaluated using ABTS and DPPH assays, revealing notable effects of both ergosterol content and processing treatment on free radical scavenging efficiency.

The FRAP assay, which quantifies the reduction of ferric (Fe^3+^) to ferrous (Fe^2+^) ions by antioxidants, confirmed the antioxidant activity of the Serpylli herba extract-loaded liposomes (Table 4). The results showed that all formulations exhibited relatively similar antioxidant capacity, ranging from ~0.13 mmol FeSO_4_/L to ~0.15 mmol FeSO_4_/L, with minor variations depending on the ergosterol content and processing conditions. Nevertheless, there were no statistically significant differences in the reducing potential of developed liposomes, suggesting that higher ergosterol content does not necessarily enhance antioxidant capacity in the FRAP assay. In fact, increased sterol concentration might result in tighter membrane packing, possibly limiting the accessibility of ferric ions to encapsulated antioxidants [30,31,40,46]. UV-irradiated liposomes did not exhibit different FRAP values compared to their non-treated counterparts. The UV-treated samples with 10 mol% and 20 mol% ergosterol showed a reducing potential of ~0.13 mmol FeSO_4_/L. Lyophilized liposomes appear to preserve antioxidant functionality in the FRAP assay, highlighting the benefit of lyophilization for stabilizing bioactives in terms of ion-reducing capacity.

The observed differences between the results from the FRAP assay and the ABTS and DPPH assays can be attributed to the distinct mechanisms underlying each antioxidant method. The FRAP assay measures the reducing power of antioxidants, specifically their ability to reduce ferric (Fe^3+^) to ferrous (Fe^2+^) ions under acidic conditions, which primarily reflects electron-donating capacity. In contrast, the ABTS and DPPH assays evaluate radical scavenging activity, reflecting both hydrogen atom transfer and single-electron transfer mechanisms in a free radical environment. As a result, certain compounds may exhibit strong reducing activity in the FRAP assay but moderate radical scavenging activity in ABTS or DPPH assays, or vice versa. Additionally, matrix effects, solubility, and the structural characteristics of polyphenols in Serpylli herba extracts may further contribute to these discrepancies. Therefore, using multiple complementary assays provides a more comprehensive assessment of the antioxidant potential of the formulations.

The results confirm that ergosterol-containing liposomes are effective carriers for preserving the radical scavenging and ion-reducing functionality of Serpylli herba polyphenols, even after exposure to post-preparation treatments. The presence of ergosterol likely enhances membrane rigidity and stability, thereby protecting encapsulated bioactives from oxidative degradation. These findings highlight the potential of such systems for the development of stable antioxidant delivery platforms for pharmaceutical or nutraceutical applications.

The TBARS assay was used to assess the capacity of Serpylli herba extract to suppress or delay lipid peroxidation triggered by UV irradiation. The method relies on the reaction of thiobarbituric acid with malondialdehyde (MDA), a secondary oxidation product derived from the breakdown of lipid hydroperoxides, producing a chromogenic complex detectable at 532 nm.

**Figure 10:**
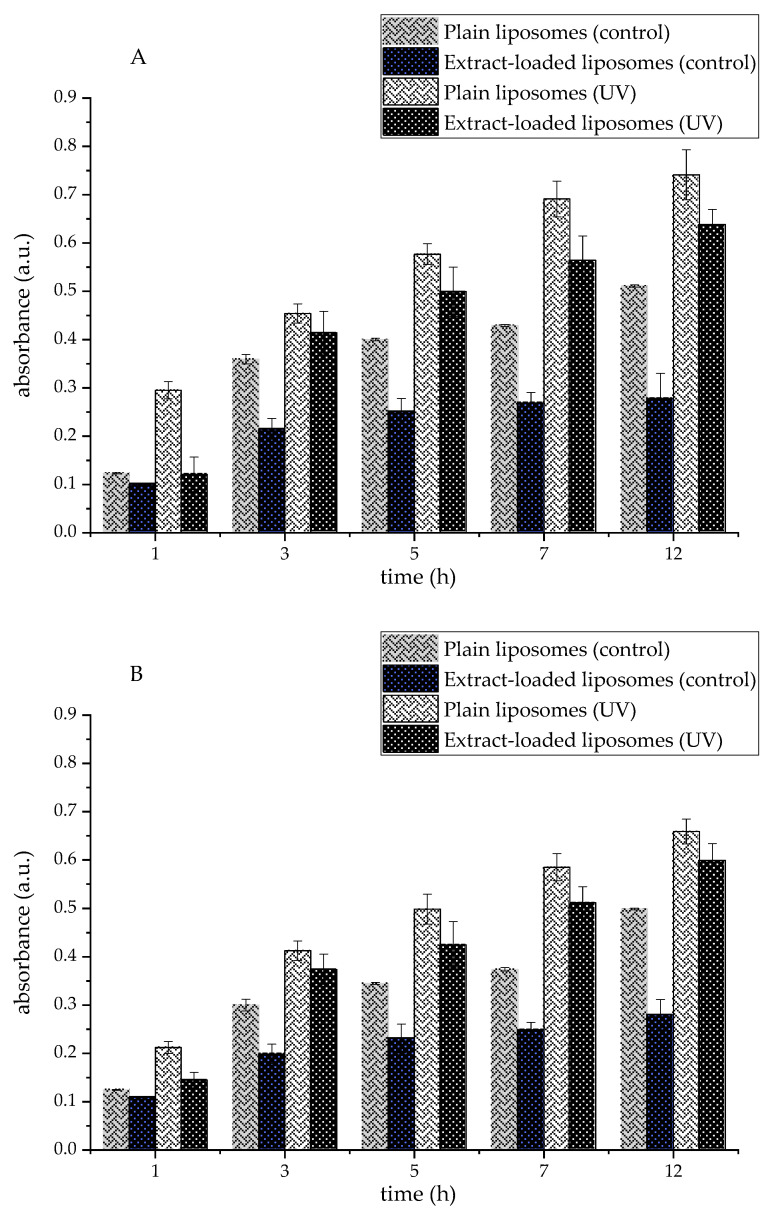
Effects of Serpylli herba extract on liposomal oxidation (thiobarbituric acid-reactive substances assay, absorbance at 532 nm) under UV irradiation (UV) and stored in the dark (control); (**A**) liposomes with 10 mol% of ergosterol, and (**B**) liposomes with 20 mol% of ergosterol.

Figure 10 summarizes the TBARS results for the different liposomal systems (unloaded and Serpylli herba extract-loaded liposomes with 10 mol% or 20 mol% of ergosterol). UV-irradiated plain liposomes containing 10 mol% or 20 mol% of ergosterol (without extract) showed a pronounced rise in MDA levels compared to extract-loaded vesicles or non-irradiated controls kept in the dark. Notably, after 5 h of UV exposure, liposomes encapsulating Serpylli herba extract exhibited markedly lower peroxidation, with the extent of protection varying according to lipid composition. These outcomes highlight the antioxidant role of Serpylli herba extract in preserving liposomal integrity and reducing oxidative damage in sterol-enriched formulations. These results align with earlier reports highlighting the capacity of polyphenols to counteract lipid peroxidation [44,100]. Nevertheless, the data shown in Figure 10 indicate that the presence of both ergosterol concentrations under UV exposure resulted in lipid oxidation. This observation agrees with our previous findings, where a concentration-dependent effect of sterols on lipid stability and peroxidation was demonstrated [40]. Such behavior may be linked to the intrinsic tendency of sterols to undergo self-oxidation, thereby accelerating oxidative damage within the lipid bilayer.

While our study did not assess the cytotoxicity of ergosterol-enriched liposomes, existing literature indicates that ergosterol exhibits low cytotoxicity at concentrations typically used in liposomal formulations. For instance, ergosterol-loaded poly(lactide-co-glycolide) nanoparticles demonstrated stronger cytotoxicity against human cancer cell lines compared to free ergosterol, in glioma U251, breast cancer MCF-7, and hepatoma HepG2 cells [101]. Additionally, ergosterol has been incorporated into liposomal formulations to enhance the delivery and efficacy of anticancer agents. For example, ergosterol and cisplatin-loaded liposomes exhibited significant inhibition of A549 lung cancer cell proliferation, with the highest cellular uptake and strongest inhibitory effect observed at 4 h, suggesting a promising drug delivery system to improve anticancer drug effects and tumor targeting in vitro [102]. These findings suggest that ergosterol, when incorporated into liposomal formulations, can enhance the therapeutic efficacy of encapsulated agents without significant cytotoxicity. Nevertheless, the cytotoxicity of ergosterol-enriched liposomes can vary depending on the concentration of ergosterol and the specific cell lines used. Therefore, future studies should include direct cytotoxicity assessments using appropriate cell lines to further confirm the biocompatibility and safety of ergosterol-enriched liposomes.

Ergosterol-containing liposomes offer a promising platform for oral or topical delivery of bioactive plant polyphenols, with potential applications in antioxidant supplements, anti-inflammatory nutraceuticals, and dermo-cosmetic formulations. Their enhanced stability and preserved bioactivity may facilitate the development of standardized, reproducible products that comply with regulatory requirements for shelf-life and quality control. Moreover, the use of ergosterol as a natural, vegan-friendly sterol aligns with current trends in sustainable formulation development. Future translational studies could include preclinical pharmacokinetic evaluation, scale-up of liposome production, and assessment of bioavailability, providing critical information for eventual regulatory approval and commercial application.

## 4. Conclusions

This study demonstrated that increasing ergosterol content enhances the encapsulation efficiency and structural resilience of Serpylli herba extract-loaded liposomes, particularly under UV irradiation and lyophilization. Liposomes with 20 mol% ergosterol showed superior polyphenol retention, more consistent vesicle size, and improved antioxidant activity compared to formulations with lower sterol content. Extract loading reduced vesicle size and zeta potential, likely due to polyphenol–lipid interactions affecting membrane packing and surface charge. UV treatment had minimal effects on liposomal size or charge, while lyophilization caused increased size and PDI, indicating temporary structural destabilization. However, ergosterol enrichment mitigated these effects, preserving membrane integrity and charge stability. Throughout 28 days of storage, extract-loaded liposomes with 20 mol% ergosterol maintained better physicochemical properties, including size, surface charge, and mobility, highlighting sterol’s role in stabilizing bilayer structure. UV-irradiated liposomes also retained their structural integrity and antioxidant function, particularly in high-ergosterol formulations. Lyophilized systems experienced early destabilization but recovered by the 28th day, with 20 mol% ergosterol liposomes showing improved reconstitution behavior. The findings of the NTA emphasize that ergosterol-enriched liposomes exhibit ergosterol concentration-dependent differences in particle numbers, which may critically influence their biological performance, release kinetics, and stability in pharmaceutical and nutraceutical applications. TEM findings confirm that ergosterol-enriched liposomes preserve structural integrity and spherical morphology, underscoring their potential as stable carriers for plant-derived bioactives. Encapsulation of Serpylli herba extract in ergosterol-based liposomes reduced the diffusion rate of polyphenols, demonstrating prolonged release capacity, although UV exposure partially compromised barrier integrity. Antioxidant activity was preserved across all formulations, though a higher ergosterol level offered greater stability. UV-treated liposomes maintained radical scavenging activity, while lyophilization preserved ABTS inhibition but reduced DPPH responsiveness, emphasizing the need for multiple antioxidant assays. FRAP results were comparable among formulations, suggesting that ergosterol may not significantly influence reducing power due to limited ferric ion access in tightly packed membranes. The findings from the TBARS assay confirm that while ergosterol-enriched liposomes are susceptible to oxidative stress under UV irradiation, co-loading with Serpylli herba extract provides effective protection against lipid peroxidation, thereby enhancing formulation stability. Nevertheless, some limitations were identified. Formulations with ergosterol content above 20 mol% became unstable due to sterol precipitation, and the absence of cryoprotectants likely contributed to size increase and partial loss of encapsulated compounds post lyophilization. Future studies should deeply explore optimal sterol concentrations beyond 20 mol%, assess the use of cryoprotectants, and evaluate the pharmacological efficacy, bioavailability, and safety of the developed systems. These findings support the use of ergosterol-enriched liposomes as promising carriers for Serpylli herba-derived antioxidants, offering improved stability and functionality under various processing and storage conditions, with potential application in pharmaceutical and nutraceutical formulations. The study also demonstrates the stabilizing potential of ergosterol in liposomes and provides a basis for future studies exploring combinations with cryoprotectants, scale-up processes, and in vivo evaluation of bioactive delivery efficiency. Future studies could also focus on directly assessing the impact of ergosterol on bilayer microviscosity and related biophysical properties to provide a more comprehensive understanding of its stabilizing role. Additionally, future studies should investigate the cytotoxicity of ergosterol-enriched liposomes across different cell lines to confirm their biocompatibility and optimize therapeutic safety.

## Figures and Tables

**Table 1:** Efficiency of encapsulation (EE) of polyphenols from Serpylli herba ethanol extract, and particle size, polydispersity index (PDI), zeta potential (ζ), and mobility (µ) of unloaded and extract-loaded phospholipid-ergosterol liposomes (10 mol% and 20 mol% of ergosterol, ergo 10% and ergo 20%, respectively), measured immediately after the preparation of liposomes (non-treated), UV irradiation and lyophilization.

Sample		EE (%)	Size (nm)	PDI	ζ (mV)	*µ* (µm·cm/V·s)
non-treated	ergo 10%	n.a.	604.7 ± 17.2 ^b^*	0.235 ± 0.005 ^d^	−27.3 ± 0.7 ^a^	−2.19 ± 0.10 ^ab^
	ergo 10% + e	75.6 ± 1.0 ^b^	460.0 ± 19.6 ^d^	0.355 ± 0.046 ^b^	−25.0 ± 0.5 ^b^	−1.57 ± 0.04 ^e^
	ergo 20%	n.a.	596.0 ± 15.6 ^b^	0.276 ± 0.023 ^c^	−24.3 ± 1.0 ^bc^	−1.89 ± 0.13 ^cd^
	ergo 20% + e	81.0 ± 0.8 ^a^	445.0 ± 29.5 ^d^	0.389 ± 0.005 ^b^	−22.5 ± 0.9 ^c^	−1.60 ± 0.04 ^e^
UV-irradiated	ergo 10%	n.a.	602.5 ± 3.5 ^b^	0.251 ± 0.010 ^c^	−25.6 ± 0.4 ^b^	−1.98 ± 0.08 ^c^
	ergo 10% + e	73.9 ± 0.5 ^c^	451.3 ± 13.0 ^d^	0.364 ± 0.023 ^b^	−23.9 ± 0.5 ^c^	−1.86 ± 0.02 ^c^
	ergo 20%	n.a.	586.0 ± 15.5 ^b^	0.255 ± 0.020 ^cd^	−25.9 ± 0.5 ^b^	−2.06 ± 0.10 ^bc^
	ergo 20% + e	81.5 ± 1.4 ^a^	454.3 ± 14.0 ^d^	0.387 ± 0.100 ^bc^	−22.6 ± 0.8 ^c^	−1.75 ± 0.06 ^d^
lyophilized	ergo 10%	n.a.	637.5 ± 4.7 ^a^	0.846 ± 0.109 ^a^	−26.8 ± 1.0 ^a^	−2.10 ± 0.07 ^bc^
	ergo 10% + e	49.4 ± 0.8 ^e^	539.7 ± 16.2 ^c^	0.857 ± 0.084 ^a^	−27.5 ± 0.4 ^a^	−2.13 ± 0.09 ^b^
	ergo 20%	n.a.	644.3 ± 5.1 ^a^	0.787 ± 0.010 ^a^	−28.9 ± 1.1 ^a^	−2.34 ± 0.06 ^a^
	ergo 20% + e	70.4 ± 0.6 ^d^	544.3 ± 12.6 ^c^	0.720 ± 0.053 ^a^	−27.3 ± 0.6 ^a^	−2.02 ± 0.06 ^bc^

* Values with different letters in each row showed statistically significant differences (*p* < 0.05; *n* = 3; analysis of variance, Duncan’s post hoc test); ergo, ergosterol, e, extract, n.a., not applicable.

**Table 2:** Density (ρ), surface tension (γ), and viscosity (η) of unloaded and Serpylli herba extract-loaded phospholipid-ergosterol liposomes (10 mol% and 20 mol% of ergosterol, ergo 10% and ergo 20%, respectively), measured immediately after the preparation of liposomes and UV irradiation.

Sample		*ρ* (g/mL)	*γ* (mN/m)	*η* (mPa·s)
non-treated	ergo 10%	0.998 ± 0.001 ^a^*	26.3 ± 1.5 ^a^	15.0 ± 0.3 ^c^
	ergo 10% + e	0.996 ± 0.003 ^a^	25.4 ± 1.6 ^a^	21.8 ± 0.5 ^a^
	ergo 20%	0.999 ± 0.002 ^a^	25.7 ± 0.5 ^a^	15.7 ± 0.5 ^c^
	ergo 20% + e	0.997 ± 0.002 ^a^	26.5 ± 0.8 ^a^	22.3 ± 0.9 ^a^
UV-irradiated	ergo 10%	1.000 ± 0.003 ^a^	24.9 ± 0.8 ^a^	11.9 ± 0.7 ^d^
	ergo 10% + e	0.996 ± 0.003 ^a^	25.0 ± 0.9 ^a^	16.6 ± 1.0 ^bc^
	ergo 20%	0.998 ± 0.002 ^a^	25.1 ± 1.2 ^a^	10.3 ± 1.1 ^d^
	ergo 20% + e	1.001 ± 0.002 ^a^	26.9 ± 1.4 ^a^	16.8 ± 0.3 ^b^

* Values with different letters in each row showed statistically significant differences (*p* < 0.05; *n* = 3; analysis of variance, Duncan’s post hoc test); ergo, ergosterol, e, extract.

**Table 3:** Diffusion coefficients (D) and diffusion resistance (R) of Serpylli herba extract and extract-loaded liposomes with 20 mol% of ergosterol (non-treated and UV-irradiated) in water.

Sample	D (m^2^/s)	R (s/m)
extract	5.76 × 10^−9^	3.54 × 10^5^
non-treated liposomes	9.37 × 10^−10^	2.17 × 10^6^
UV-irradiated liposomes	1.81 × 10^−9^	1.13 × 10^6^

**Table 4:** Antioxidant capacity of Serpylli herba extract-loaded liposomes (non-treated, UV-irradiated, and lyophilized) obtained in the FRAP assay.

Sample		FRAP (mmol FeSO_4_/L)
non-treated	ergo 10% + e	0.14 ± 0.00 ^a^*
	ergo 20% + e	0.15 ± 0.02 ^a^
UV-irradiated	ergo 10% + e	0.13 ± 0.01 ^a^
	ergo 20% + e	0.13 ± 0.02 ^a^
lyophilized	ergo 10% + e	0.14 ± 0.02 ^a^
	ergo 20% + e	0.13 ± 0.02 ^a^

* Values with the same letters showed no statistically significant differences (*p* > 0.05; *n* = 3; analysis of variance, Duncan’s post hoc test; mean ± SD); ergo, ergosterol; e, extract.

## Data Availability

The datasets generated during and/or analyzed during the current study are available from the corresponding author upon request.

## Supplementary Materials

The following supporting information can be downloaded at: https://www.mdpi.com/article/10.3390/pharmaceutics17111362/s1, Table S1. 95% confidence intervals (CI) for entrapment efficiency (EE), particle size, polydispersity index (PDI), zeta potential (ζ), and electrophoretic mobility (µ) of ergosterol-containing liposomal formulations with Serpylli herba extract; ergo—ergosterol, e—extract; Table S2. 95% confidence intervals (CI) for density (ρ), surface tension (γ), and viscosity (η) of ergosterol-containing liposomal formulations with Serpylli herba extract; ergo—ergosterol, e—extract; Table S3. 95% confidence intervals (CI) for results from ABTS, DPPH, and FRAP assays of the antioxidant potential of ergosterol-containing liposomal formulations with Serpylli herba extract; ergo—ergosterol, e—extract; Figure S1. (A) Particle size—bars and polydispersity index—numbers above bars, and (B) zeta potential—bars and mobility—numbers above bars (µm·cm/V·s) of unloaded liposomes with 10 mol% or 20 mol% of ergosterol (non-treated samples) for 28 days of storage at 4 °C; ergo, ergosterol; Figure S2. (A) Particle size—bars and polydispersity index—numbers above bars, and (B) zeta potential—bars and mobility—numbers above bars (µm·cm/V·s) of unloaded liposomes with 10 mol% or 20 mol% of ergosterol (UV-irradiated samples) for 28 days of storage at 4 °C; ergo, ergosterol; Figure S3. (A) Particle size—bars and polydispersity index—numbers above bars, and (B) zeta potential—bars and mobility—numbers above bars (µm·cm/V·s) of unloaded liposomes with 10 mol% or 20 mol% of ergosterol (lyophilized samples) for 28 days of storage at 4 °C; ergo, ergosterol; Figure S4. Dimensionless plot of polyphenol concentration vs. time for the curves of polyphenol release in water using a Franz diffusion cell.
