# Supplementary material for: Ergosterol-Enriched Liposomes with Post-Processing Modifications for Serpylli Herba Polyphenol Delivery: Physicochemical, Stability and Antioxidant Assessment
Source: Pharmaceutics. 2025 Oct 22;17(11):1362. doi: 10.3390/pharmaceutics17111362 (PMC12655308; doi:10.3390/pharmaceutics17111362)
Supplement: Supplementary file 1 [file pharmaceutics-17-01362-s001.zip › pharmaceutics-3897765-supplementary.pdf]

pharmaceutics

MDPI

Ergosterol-Enriched Liposomes with Post-Processing Modifications for Serpylli Herba Polyphenol Delivery: Physicochemical, Stability and Antioxidant Assessment

Aleksandra A. Jovanović [1], Predrag Petrović [2], Andrea Pirković [1], Ninoslav Mitić [1], Francesca Giampieri [3,4,5], Maurizio Battino [3,4,5,6,*] and Dragana Dekanski [1]

Supplementary material

Table S1. 95% confidence intervals (CI) for entrapment efficiency (EE), particle size, polydispersity index (PDI), zeta potential (ζ), and electrophoretic mobility (μ) of ergosterol-containing liposomal formulations with Serpylli herba extract; ergo – ergosterol, e - extract.

sample		95% CI for EE (%)	95% CI for size (nm)	95% CI for PDI	95% CI for ζ (mV)	95% CI for μ (μm·cm/V·s)
non-treated	ergo 10%	n.a.	562.0-647.4	0.223-0.247	-29.0– -25.6	-2.438– -1.942
	ergo 10%+e	73.1-78.1	411.4-508.6	0.241-0.469	-26.2– -23.8	-1.669– -1.471
	ergo 20%	n.a.	557.3-634.7	0.219-0.333	-26.8– -21.8	-2.213– -1.567
	ergo 20%+e	79.0-83.0	371.8-518.2	0.376-0.401	-24.7– -20.3	-1.699– -1.501
UV-irradiated	ergo 10%	n.a.	593.8-611.2	0.226-0.276	-26.6– -24.6	-2.179– -1.781
	ergo 10%+e	72.7-75.1	419.0-483.6	0.307-0.421	-25.1– -22.7	-1.910– -1.81
	ergo 20%	n.a.	547.5-624.5	0.205-0.305	-27.1– -24.7	-2.308– -1.812
	ergo 20%+e	78.0-85.0	419.6-489.0	0.139-0.635	-24.6– -20.6	-1.899– -1.601
lyophilized	ergo 10%	n.a.	625.9-649.1	0.575-1.117	-29.3– -24.3	-2.274– -1.926
	ergo 10%+e	47.4-51.4	499.5-579.9	0.649-1.065	-28.5– -26.5	-2.354– -1.906
	ergo 20%	n.a.	631.7-656.9	0.762-0.812	-31.6– -26.2	-2.489– -2.191
	ergo 20%+e	68.9-71.9	513.0-575.6	0.589-0.851	-28.8– -25.8	-2.169– -1.871

Table S2. 95% confidence intervals (CI) for density (ϱ), surface tension (γ), and viscosity (η) of ergosterol-containing liposomal formulations with Serpylli herba extract; ergo – ergosterol, e - extract.

sample		95% CI for ρ (g/mL)	95% CI for γ (mN/m)	95% CI for η (mPa·s)
non-treated	ergo 10%	0.9955-1.0005	22.60-30.00	14.26-15.74
	ergo 10%+e	0.9885-1.0035	21.43-29.37	20.56-23.04
	ergo 20%	0.9940-1.0040	24.46-26.94	14.46-16.94
	ergo 20%+e	0.9920-1.0020	24.51-28.49	20.07-24.53
UV-irradiated	ergo 10%	0.9925-1.0075	22.91-26.89	10.16-13.64
	ergo 10%+e	0.9885-1.0035	22.77-27.23	14.12-19.08
	ergo 20%	0.9930-1.0030	22.12-28.08	7.57-13.03
	ergo 20%+e	0.9960-1.0060	23.43-30.37	16.06-17.54

Table S3. 95% confidence intervals (CI) for results from ABTS, DPPH, and FRAP assays of the antioxidant potential of ergosterol-containing liposomal formulations with Serpylli herba extract; ergo – ergosterol, e - extract.

**sample**		**95% CI for ABTS (%)**	**95% CI for DPPH (%)**	**95% CI for FRAP (mmol $FeSO_4$/L)**
non-treated	ergo 10%+e	59.82-91.14	54.7-67.34	0.140-0.140
	ergo 20%+e	66.00-96.58	65.78-70.24	0.115-0.185
UV-irradiated	ergo 10%+e	73.78-78.74	51.95-71.93	0.105-0.155
	ergo 20%+e	71.55-93.55	55.62-76.70	0.095-0.165
lyophilized	ergo 10%+e	81.48-90.52	32.37-56.47	0.090-0.190
	ergo 20%+e	79.34-84.30	46.63-67.25	0.090-0.170

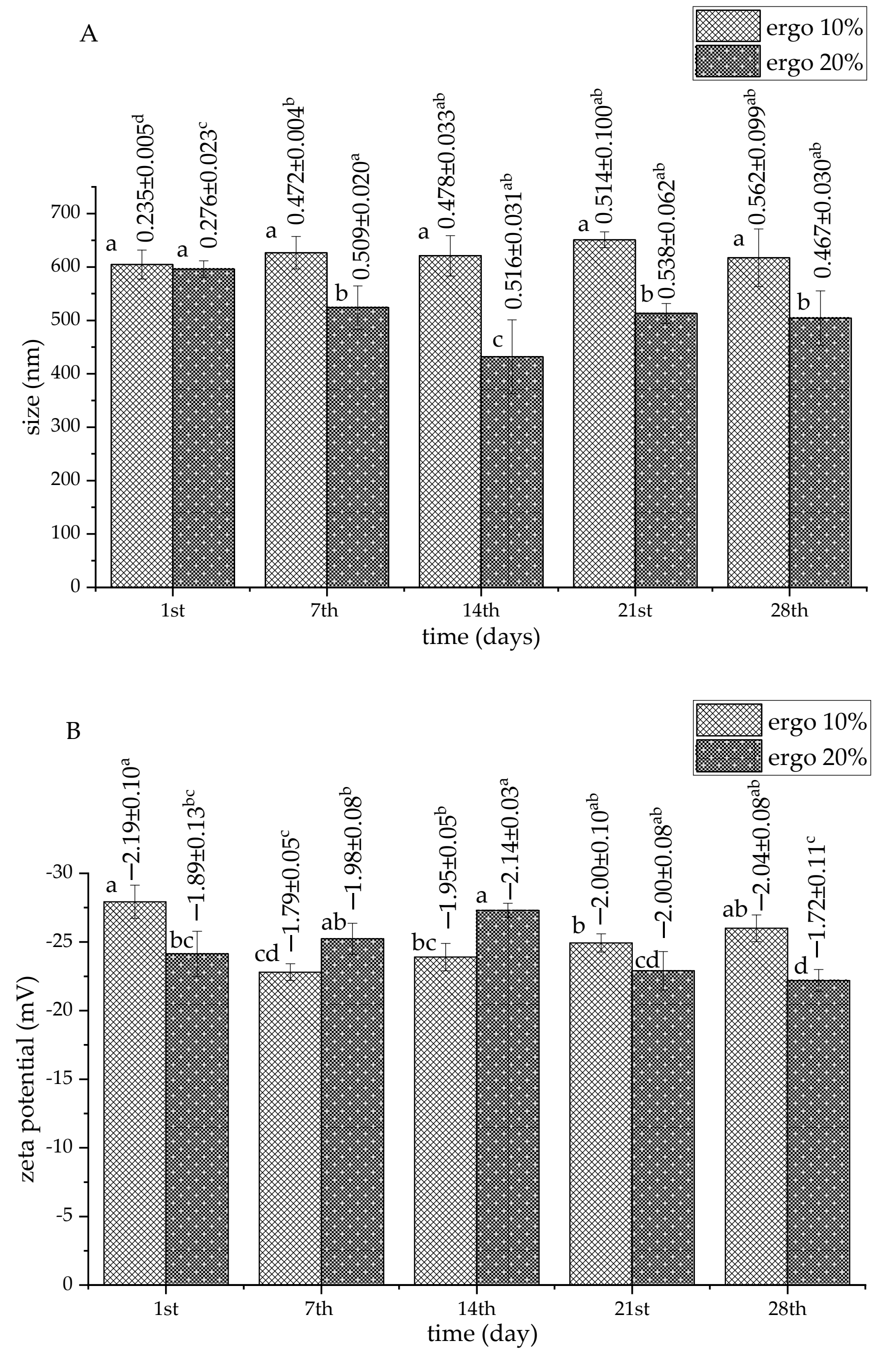

Figure S1. (A) Particle size - bars and polydispersity index - numbers above bars, and (B) zeta potential - bars and mobility - numbers above bars (μm·cm/V·s) of unloaded liposomes with 10 mol% or 20 mol% of ergosterol (non-treated samples) for 28 days of storage at 4°C; ergo, ergosterol.

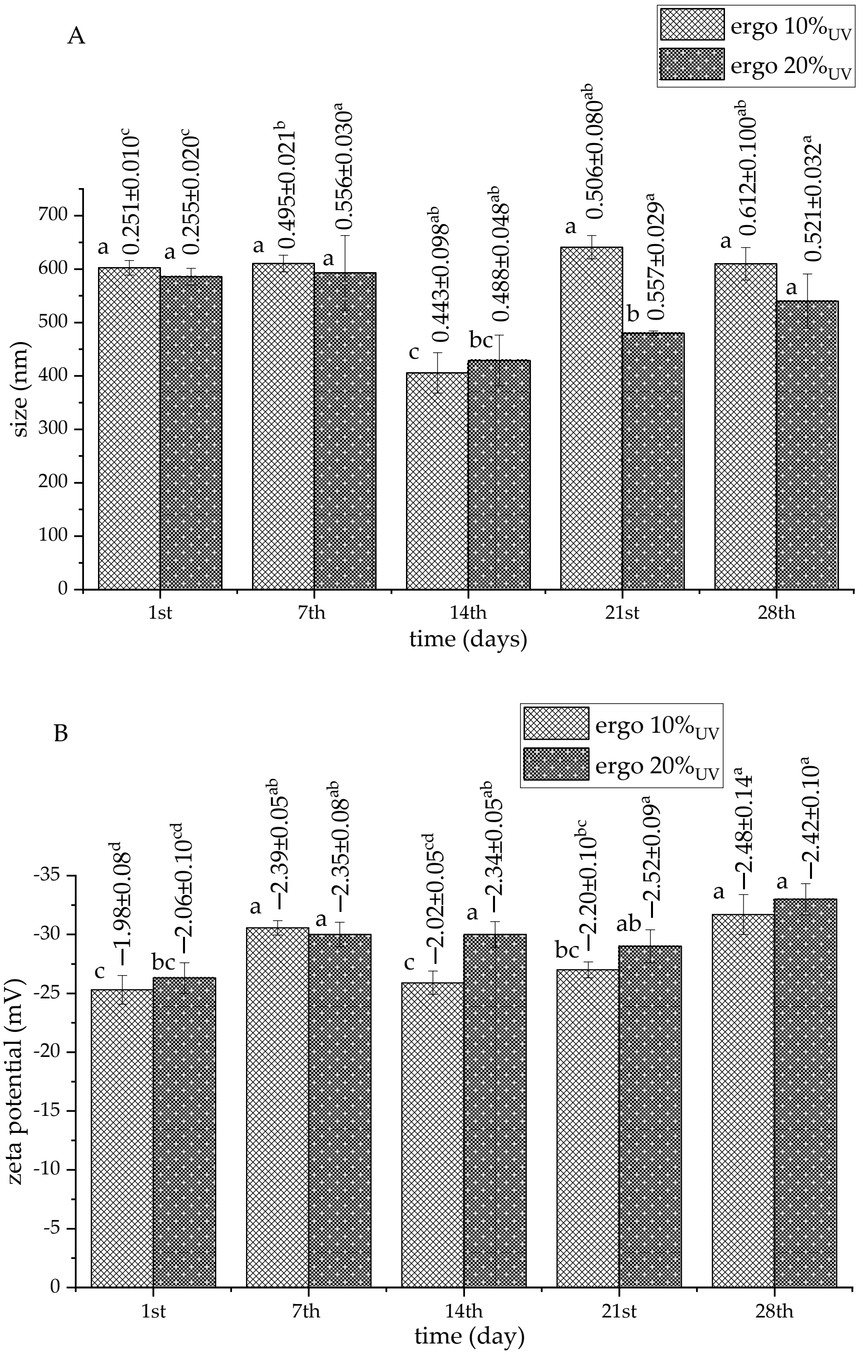

Figure S2. (A) Particle size - bars and polydispersity index - numbers above bars, and (B) zeta potential - bars and mobility - numbers above bars (μm·cm/V·s) of unloaded liposomes with 10 mol% or 20 mol% of ergosterol (UV-irradiated samples) for 28 days of storage at 4°C; ergo, ergosterol.

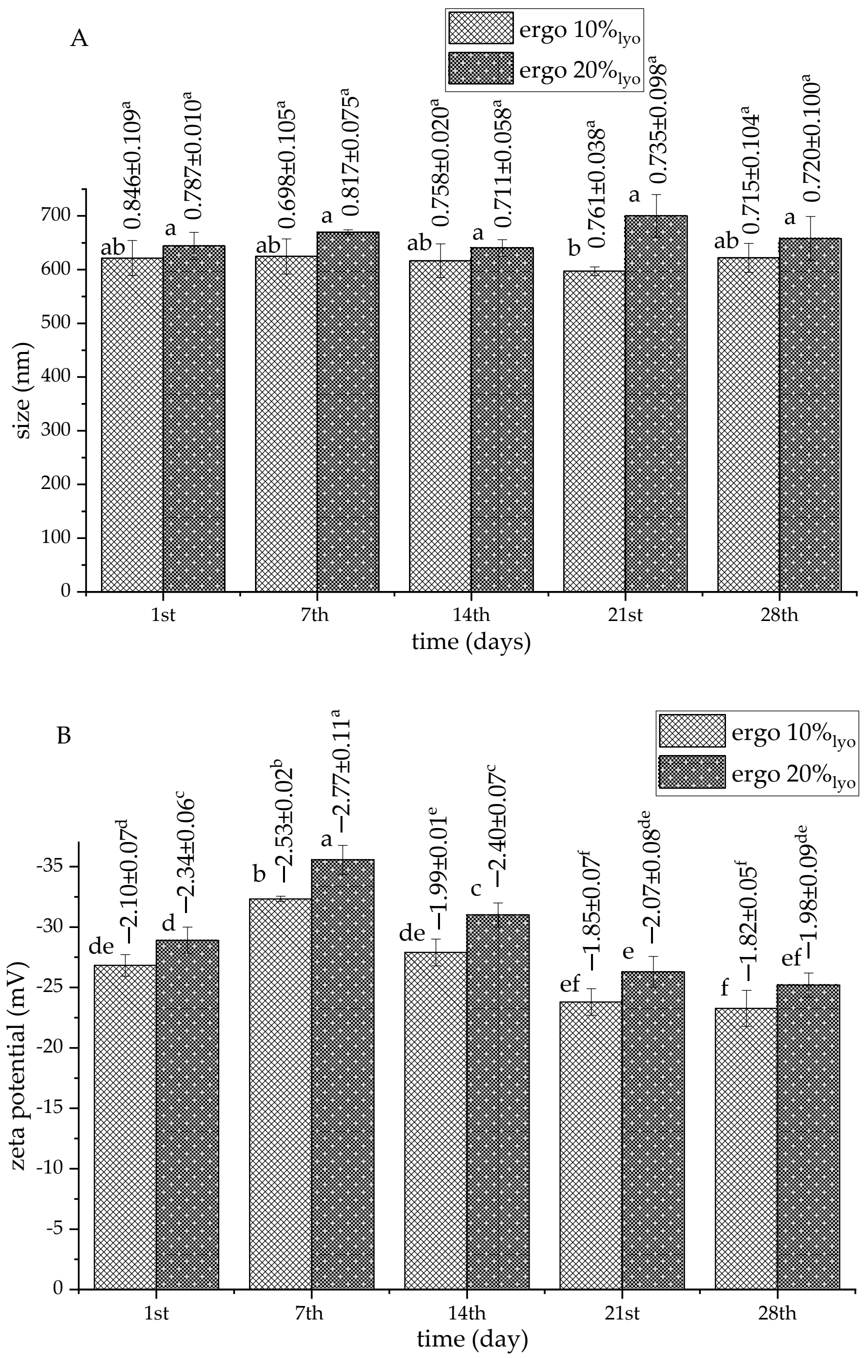

Figure S3. (A) Particle size - bars and polydispersity index - numbers above bars, and (B) zeta potential - bars and mobility - numbers above bars (μm·cm/V·s) of unloaded liposomes with 10 mol% or 20 mol% of ergosterol (lyophilized samples) for 28 days of storage at 4°C; ergo, ergosterol.

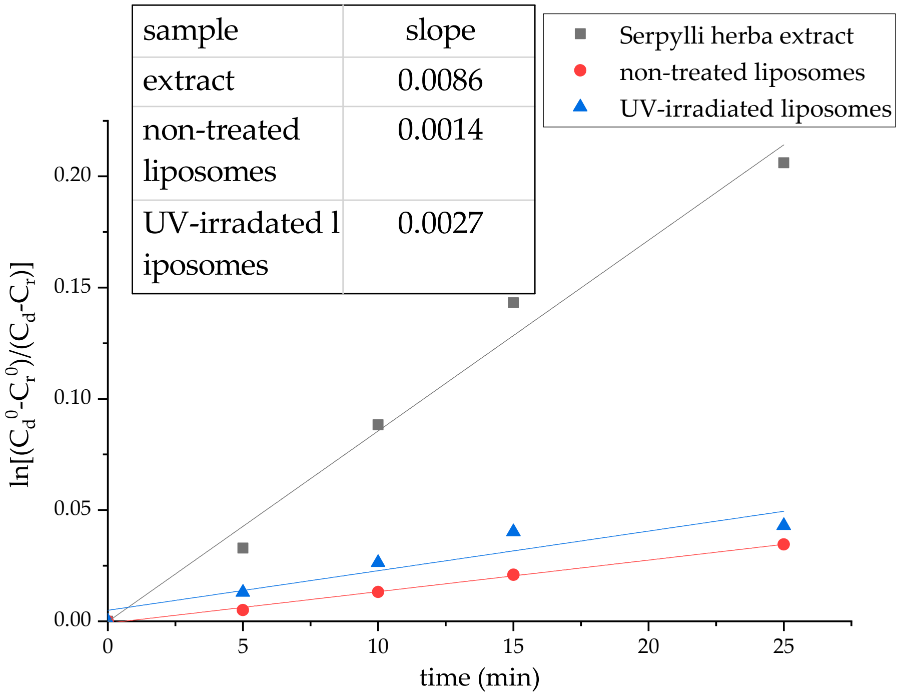

Figure S4. Dimensionless plot of polyphenol concentration *vs*. time for the curves of polyphenol release in water using a Franz diffusion cell.
